# Identification of 12 immune-related lncRNAs and molecular subtypes for the clear cell renal cell carcinoma based on RNA sequencing data

**DOI:** 10.1038/s41598-020-71150-3

**Published:** 2020-09-02

**Authors:** Weimin Zhong, Bin Chen, Hongbin Zhong, Chaoqun Huang, Jianqiong Lin, Maoshu Zhu, Miaoxuan Chen, Ying Lin, Yao Lin, Jiyi Huang

**Affiliations:** 1 The Fifth Hospital of Xiamen, Xiamen, 361101 Fujian Province People’s Republic of China; 2 Key Laboratory of Optoelectronic Science and Technology for Medicine of Ministry of Education, College of Life Sciences, Qishan Campus, Fujian Normal University, Fuzhou, 350117 Fujian Province People’s Republic of China; 3 Xiang’an Branch, The First Affiliated Hospital of Xiamen University, Xiamen University, Xiamen, 361101 Fujian Province People’s Republic of China; 4 The First Affiliated Hospital of Xiamen University, Xiamen University, Xiamen, 361003 Fujian Province People’s Republic of China

**Keywords:** Computational biology and bioinformatics, Immunology, Biomarkers, Oncology, Risk factors

## Abstract

Clear cell renal cell carcinoma (ccRCC) is the most common type of renal cell carcinoma (RCC). Despite the existing extensive research, the molecular and pathogenic mechanisms of ccRCC are elusive. We aimed to identify the immune-related lncRNA signature and molecular subtypes associated with ccRCC. By integrating 4 microarray datasets from Gene Expression Omnibus database, we identified 49 immune-related genes. The corresponding immune-related lncRNAs were further identified in the TCGA dataset. 12-lncRNAs prognostic and independent signature was identified through survival analysis and survival difference between risk groups was further identified based on the risk score. Besides, we identified 3 molecular subtypes and survival analysis result showed that cluster 2 has a better survival outcome. Further, ssGSEA enrichment analysis for the immune-associated gene sets revealed that cluster 1 corresponded to a high immune infiltration level. While cluster 2 and cluster 3 corresponded to low and medium immune infiltration level, respectively. In addition, we validated the 12-lncRNA prognostic signature and molecular subtypes in an external validation dataset from the ICGC database. In summary, we identified a 12-lncRNA prognostic signature which may provide new insights into the molecular mechanisms of ccRCC and the molecular subtypes provided a theoretical basis for personalized treatment by clinicians.

## Introduction

Renal cell carcinoma (RCC) accounts for over 90% of all kidney cancer cases among human adults^1^. Clear cell renal cell carcinoma (ccRCC) is the most common RCC histological subtype which responsible for 70–80% of RCC cases^2^. Recent studies estimate that 102,000 patients succumb to ccRCC and 202,000 new cases are diagnosed annually throughout the globe^3^. Besides, ccRCC has no clear symptoms in its early stages therefore can only be diagnosed in advanced stages^4^. Despite interventions through chemotherapy and radiotherapy, ccRCC is prone to distant metastasis. These metastases occur in lung, bone, liver, distant lymph nodes, and renal vein^5^. A few effective biomarkers, particularly in both early and advanced stages of ccRCC, have been identified.

The recent version of the human genome annotation transcribes approximately 16,000 long non-coding RNAs (lncRNAs) responsible for 26.7% of the total genes (https://www.gencodegenes.org/human/)^6^. lncRNAs are non-coding RNAs composed of about 200 nucleotides in length. They include the antisense lncRNAs, intronic transcripts, large intergenic noncoding RNAs (lincRNAs), promoter-associated lncRNAs, and UTR associated lncRNAs^7^. Previous studies have revealed that lncRNA plays an important role in regulating gene expression, epigenetics, cell differentiation, and ontogeny^8,9^. Additionally, studies have shown that massive aberrantly expressed lncRNAs are highly linked to tumor diagnosis and metastasis^10^. However, little information has been pronounced on the possible effects of dysregulated lncRNAs on ccRCC. Therefore, more specific and reliable lncRNA-based biomarkers need to be uncovered. This will thereby improve the prognostic accuracy of ccRCC.

This study assessed the immune-related lncRNA by integrating the GEO dataset, TCGA dataset, and ICGA dataset. We identified 12 lncRNA prognostic signature associated with ccRCC survival. Also, 3 molecular subtypes (cluster 1, cluster 2, and cluster 3) based on the immune lncRNA and gene expression profile were identified. The three molecular subtypes were highly correlated with immunity. The identification of immune-related lncRNA and molecular subtypes of ccRCC will advance the treatment of ccRCC patients, and provide a better understanding of the underlying molecular mechanisms of ccRCC.

## Results

### Identifying immune-related DEGs in ccRCC microarray datasets

**Figure 1:**
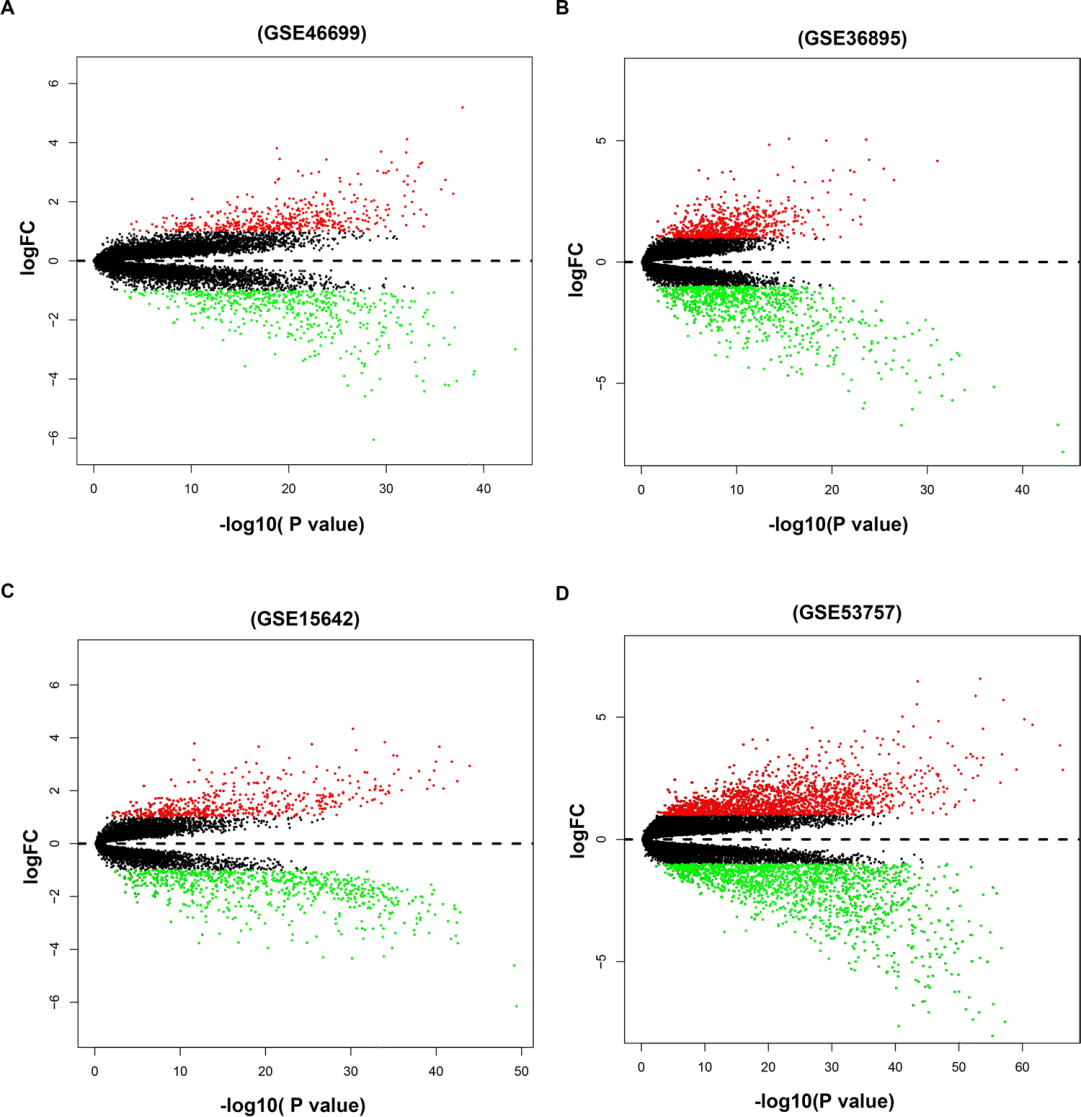
Volcano plots for the aberrantly expressed genes between ccRCC tissues and adjacent normal tissues in the four datasets. (**A**) GSE46699, (**B**) GSE36895, (**C**) GSE15642 and (**D**) GSE53757. The red dots represented up-regulated genes with the cut-off logFC > 1.0 and adjusted P < 0.05. The green dots represented down-regulated genes based on the criterion: logFC < -1.0 and adjusted P < 0.05. Black dots represent the genes have no significance.

Four microarray datasets, including GSE46699, GSE36895, GSE15641, and GSE53757 were retrieved from the GEO database. Differentially expressed analysis was conducted on these datasets. Results showed that the GSE46699 dataset had a total of 1,006 DEGs (484 up-regulated and 522 down-regulated DEGs). In the GSE36895 dataset, 725 up-regulated genes and 870 down-regulated genes were identified. Besides, in the GSE15641 dataset, there were 517 up-regulated genes and 703 down-regulated genes were identified. In addition, 1,689 up-regulated genes and 1585 down-regulated genes were identified in theGSE53757 dataset. The results DEGs from four datasets are shown in Fig. 1. A total of 326 DEGs were identified, including 145 up-regulated and 181 down-regulated DEGs through the integrating of the four datasets using the RRA algorithm (Supplementary Table 1). The top 20 up-regulated DEGs and top 20 down-regulated DEGs are highlighted in Supplementary Fig. 1.

### Identifying immune-associated lncRNAs

Among the 326 DEGs in the four datasets, 49 immune genes were identified and further extracted from the corresponding gene expression in the TCGA dataset. Accordingly, all information on the expression of lncRNAs was extracted from the TCGA dataset. To obtain high-quality lncRNA, we excluded the lowly expressed lncRNAs with the cut-off criteria that average expression in all samples need more than 0.5. The Pearson coefficient was adopted to estimate the correlation between lncRNA and immune genes. Finally, a total of 140 immune-related lncRNAs were identified based on the criterion: |cor|_pearson_ > 0.5 and p-value < 0.001 (Supplementary Table 2).

### Survival prediction for the lncRNAs and GSEA enrichment analysis

In total, 512 ccRCC patient samples with 140 immune-related lncRNAs were randomly divided into the testing dataset (N = 256) and training dataset (N = 256) respectively. The correlation of immune-related lncRNA with patients’ overall survival time was determined via univariate cox regression analysis in the training dataset. Further, we selected 56 immune-related lncRNAs with p-value < 0.05 for Lasso-penalized multivariate Cox proportional hazards modeling analysis. After 1,000 iterations, 12 immune-related lncRNA expression signatures were identified. They include: AC005104.1, AC093278.2, AC098484.1, AL360181.2, EMX2OS, LINC01011, SPINT1-AS1, AP001372.2, AC007637.1, AL354733.3, AP001189.3 and LINC00886. Moreover, these selected signatures displayed optimal survival prediction in the training dataset for more than 50 times. Further, a calculation of the 12-lncRNA signature risk score for each patient in the training and testing datasets was conducted using the risk formula:

$$\begin{aligned} {\text{Risk score}} & = 0.062557657*{\text{AC}}005104.1 + ( - 0.093367264)*{\text{AC}}093278.2 \\ & \quad + ( - 0.263904071)*{\text{AC}}098484.1 + 0.048384794*{\text{AL36}}0181.2 + ( - 0.044967607) \\ & \quad *\,{\text{EMX2OS}} + 0.23110957*{\text{LINC}}01011 + ( - 0.134140912 )*{\text{SPINT1}} - {\text{AS1}} \\ & \quad + ( - 0.063359846)*{\text{AP}}001372.2 + ( - 0.218305812 )*{\text{AC}}007637.1 + 0.178920045 \\ & \quad *{\text{AL}}354733.3 + ( - 0.074645356)*{\text{AP}}001189.3 +( - 0.220170832)*{\text{LINC}}00886. \\ \end{aligned}$$

**Figure 2:**
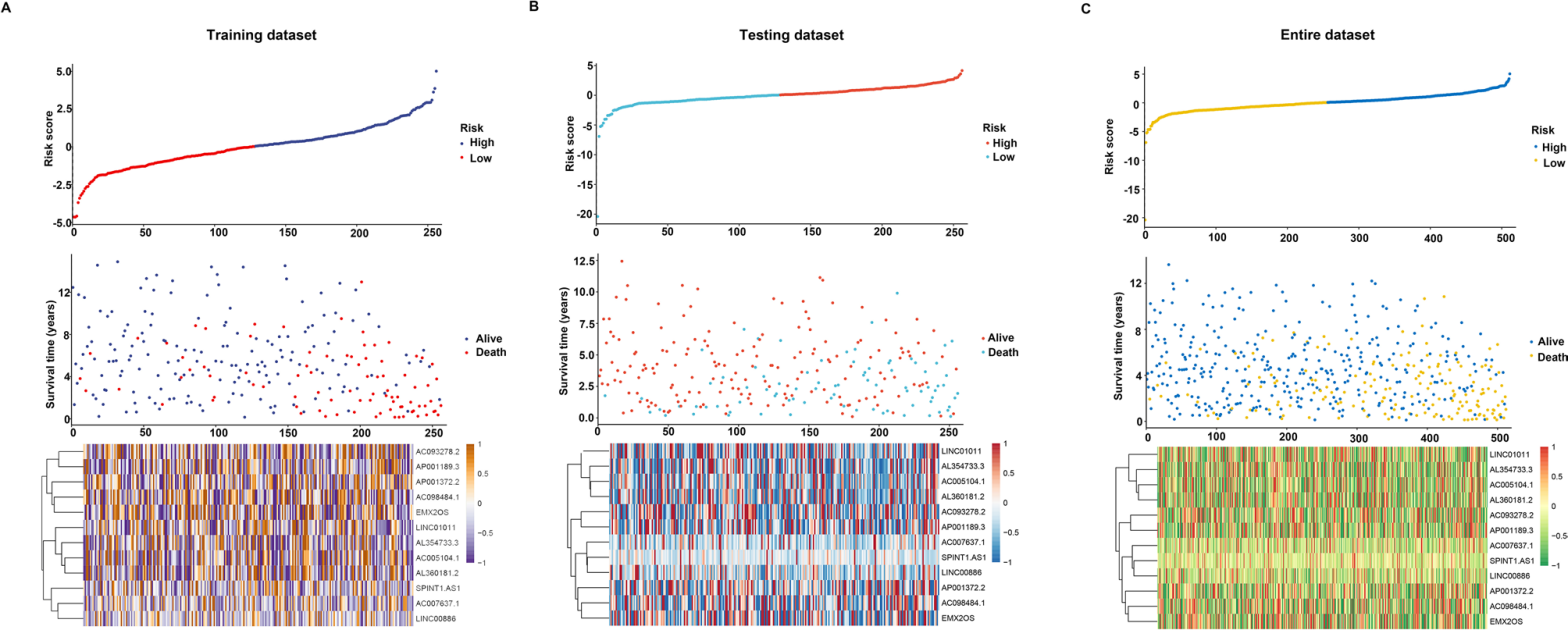
Risk plot for the ccRCC patients. (**A**) Entire dataset (**B**) Training dataset. (**C**) Testing dataset. Each panel consists of three rows: top rows showed a risk score distribution for the high risk score group and low risk score group; middle rows represent the ccRCC patients distribution and survival status; the bottom rows showed that the heatmap of 12 prognostic lncRNA expression.

**Figure 3:**
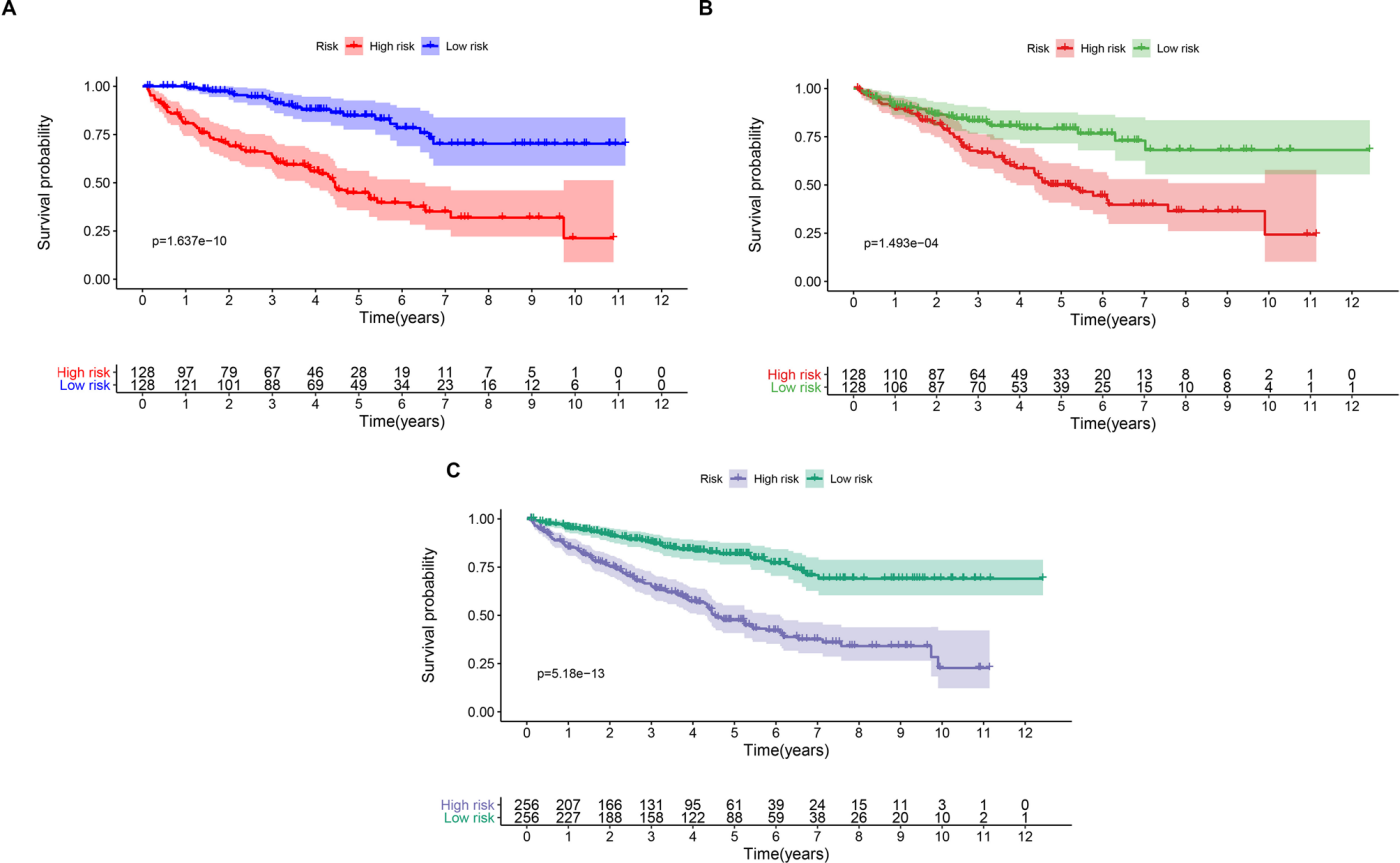
Kaplan–Meier curves for the 12‑lncRNA signature in the entire (**A**), training (**B**), and validation sets (**C**).

**Figure 4:**
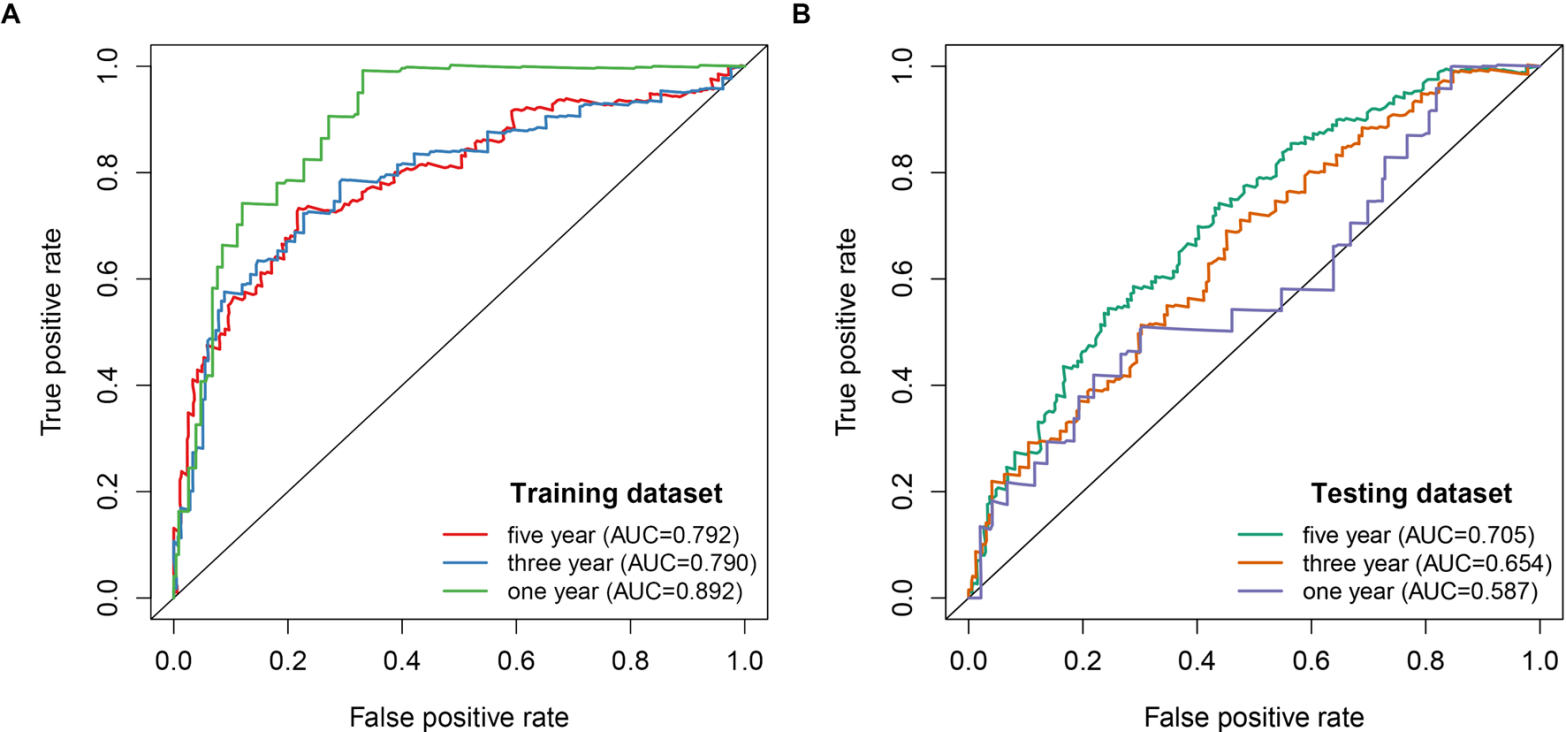
Time ROC curves analysis for the 12-lncRNA signature in the training dataset (**A**) and validation dataset (**B**).

Thus, the patients were further classified into high-risk (N = 128) and low-risk groups (N = 128). This was done according to the median risk score in the training, testing, and entire datasets respectively. The patients in the training dataset with a high-risk score corresponding to more deceased cases whereas those with low-risk scores exhibited prolonged survival time (Fig. 2A). Similar results were reported in the testing and entire datasets respectively (Fig. 2B,C). The KM curves and log-rank test identified a significant difference between the high-risk group and low-risk group from all datasets (p-value < 0.001) (Fig. 3). ROC analysis revealed that the 12-lncRNA prognostic model effectively predicted the survival of ccRCC patients in 1-year, 3-year, and 5-year period in the training dataset and testing dataset respectively (Fig. 4A,B). To further evaluate the reliability of the prognostic model, we used an external dataset from the ICGC database for verification. The risk score for each patient was calculated using the KM curve and the results which depicted a significant divergence between risk groups (Supplementary Fig. 2). Also, the ROC curve analysis showed a good performance for the risk model (Supplementary Fig. 3).

**Figure 5:**
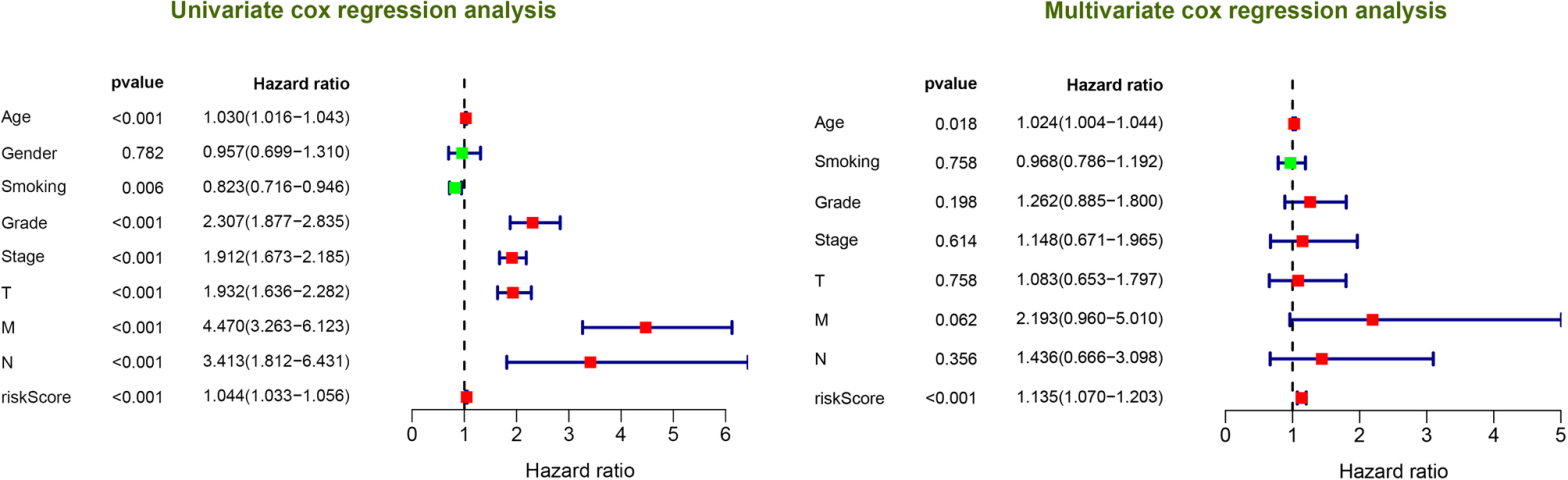
Univariate and multivariate Cox regression analysis for the 12 lncRNAs signature and clinical features.

In addition, through cox regression analysis the 12-lncRNA signature independently predicted the clinical traits (Fig. 5). To compare the accuracy of the survival prediction between 12-lncRNA signature risk models and clinical traits, we conducted a ROC analysis and observed that our risk model was optimal with an AUC value of up to 0.783 (Supplementary Fig. 4). Moreover, we investigate the association between 12 lncRNAs expression level and clinical trait (Stage and Grade), as highlighted in Supplementary Fig. 5A. The findings revealed that increased expression level of AC005104.1 and AL360181.2 from stage I to stage IV. On the other hand, the expression levels of AC093278.2, AC098484.1, and EMX2OS were decreased from stage I to stage IV. Moreover, based on the grade, expression levels of AC007637.1, AC093278.2, AC098484.1, AL354733.3, AP001189.3, AP001372.2, and EMX2OS decreased from grade 1 to grade 4 (Supplementary Fig. 5B).

**Figure 6:**
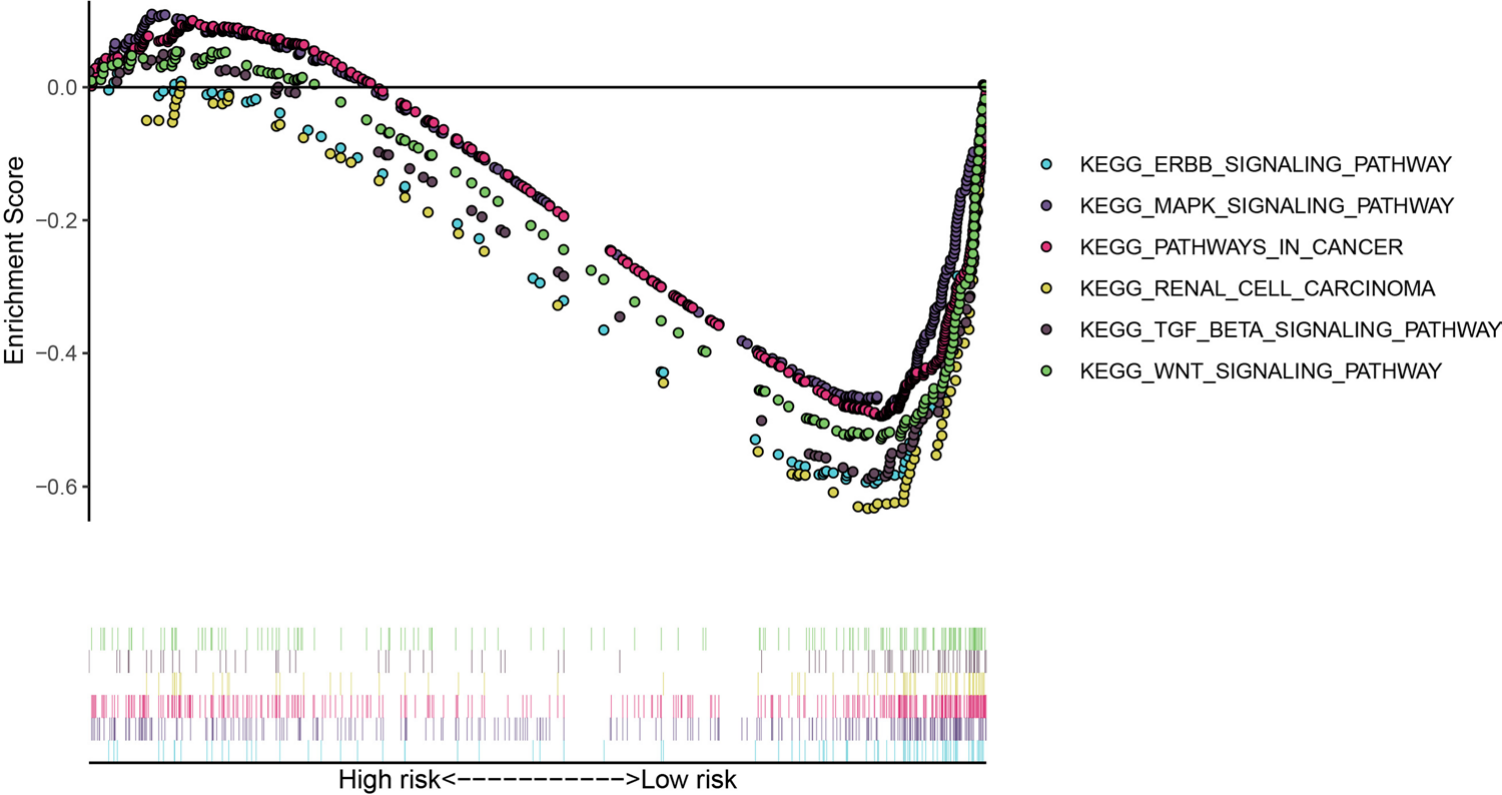
Gene Set Enrichment Analysis (GSEA) identified cancer-related pathway for the low risk group.

Through GSEA enrichment analysis, we explored the potential underlying pathway in the ccRCC in the high-risk group and low-risk group. Notably, 71 significant pathways were enriched in the low low-risk group following the cut-off: FDR < 0.05 and nominal P < 0.05. Among them, the ERBB signaling pathway, MAPK signaling pathway, pathways of cancer, renal cell carcinoma, TCG beta signaling pathway, and WNT signaling pathway were enriched in the low-risk group (Fig. 6). In contrast, there was no significantly enriched signal pathway in the high-risk group with a similar cut-off.

### Identifying molecular subtypes of ccRCC

**Figure 7:**
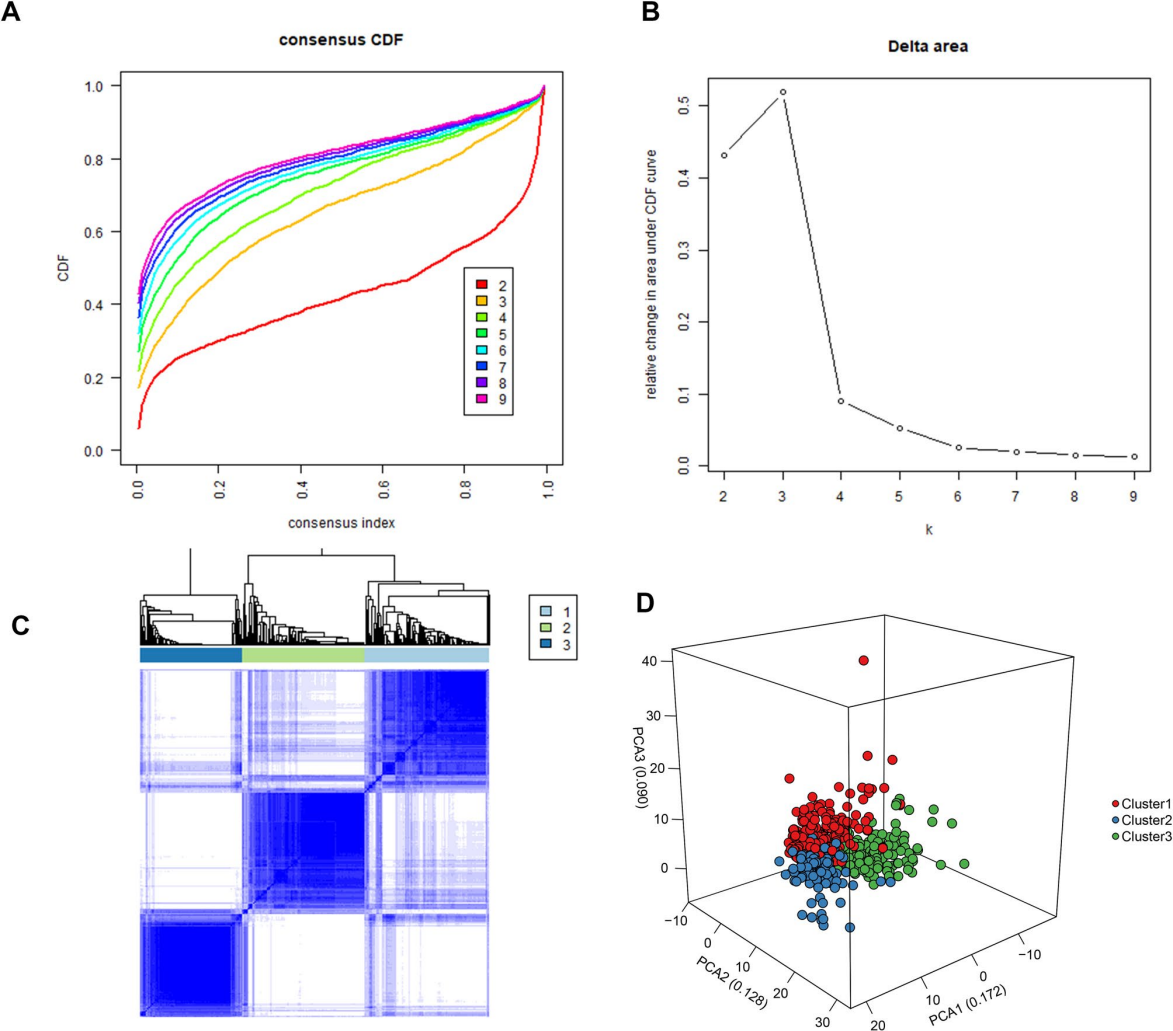
Consensus clustering for the ccRCC immune genes and immune related lncRNA expression in the TCGA dataset. (**A**) Consensus clustering cumulative distribution function (CDF) for k = 2 to 9. (**B**) The Relative change in area under CDF curve for k = 2 to 9. (**C**) Consensus matrix heatmap plots when k = 3. (**D**) Principal component analysis of the immune genes and lncRNAs expression when k = 3.

**Figure 8:**
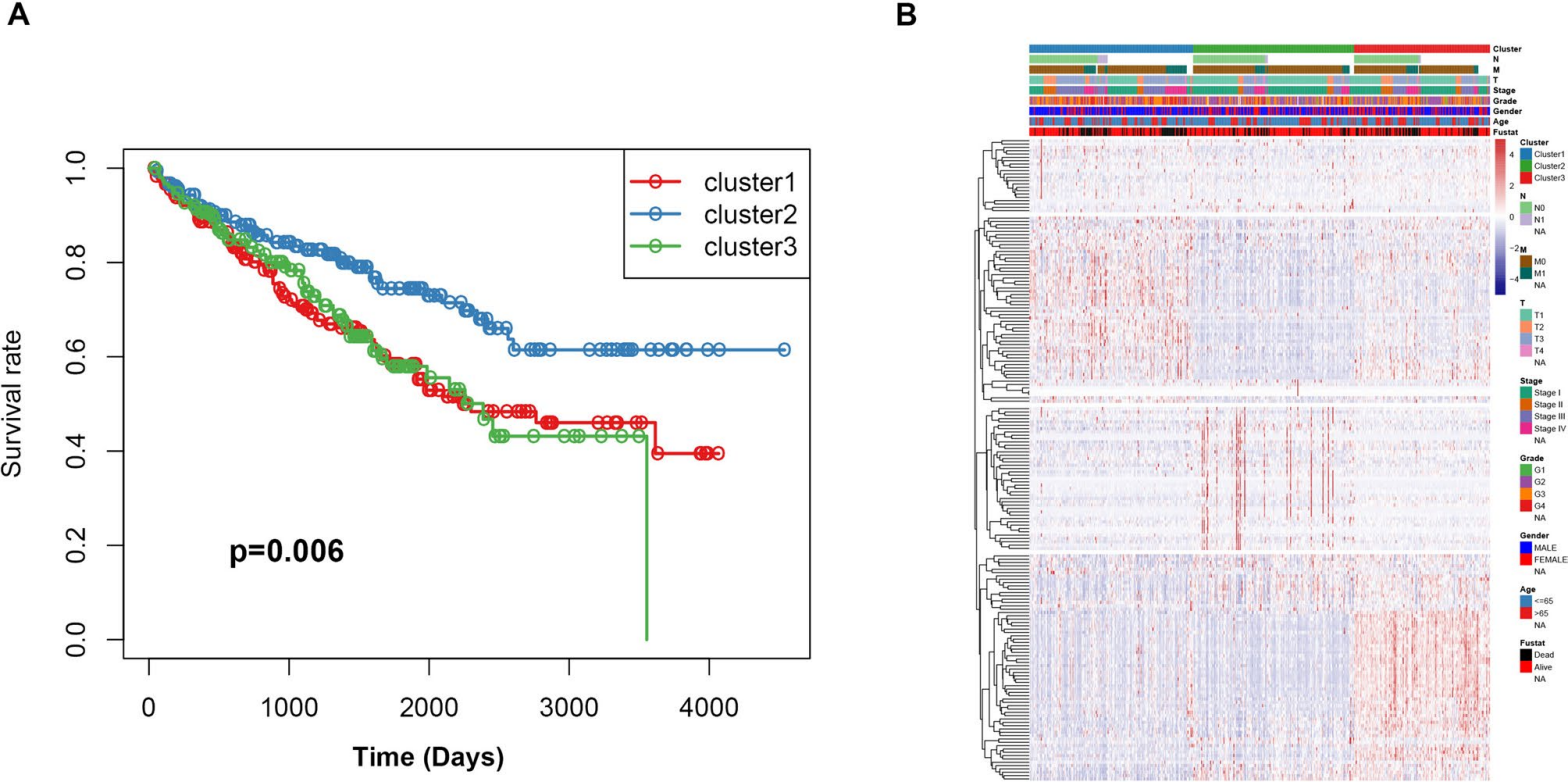
Prognostic significance of ccRCC immune subtypes. (**A**) Kaplan–Meier curves for the overall survival of different subtypes. The cluster 2 is associated with better outcomes compared to cluster 1 and cluster 3. (**B**) Heatmap of the immune genes and immune related lncRNAs expression profiles ordered by three subtypes, with clinical features associated with each cluster.

**Figure 9:**
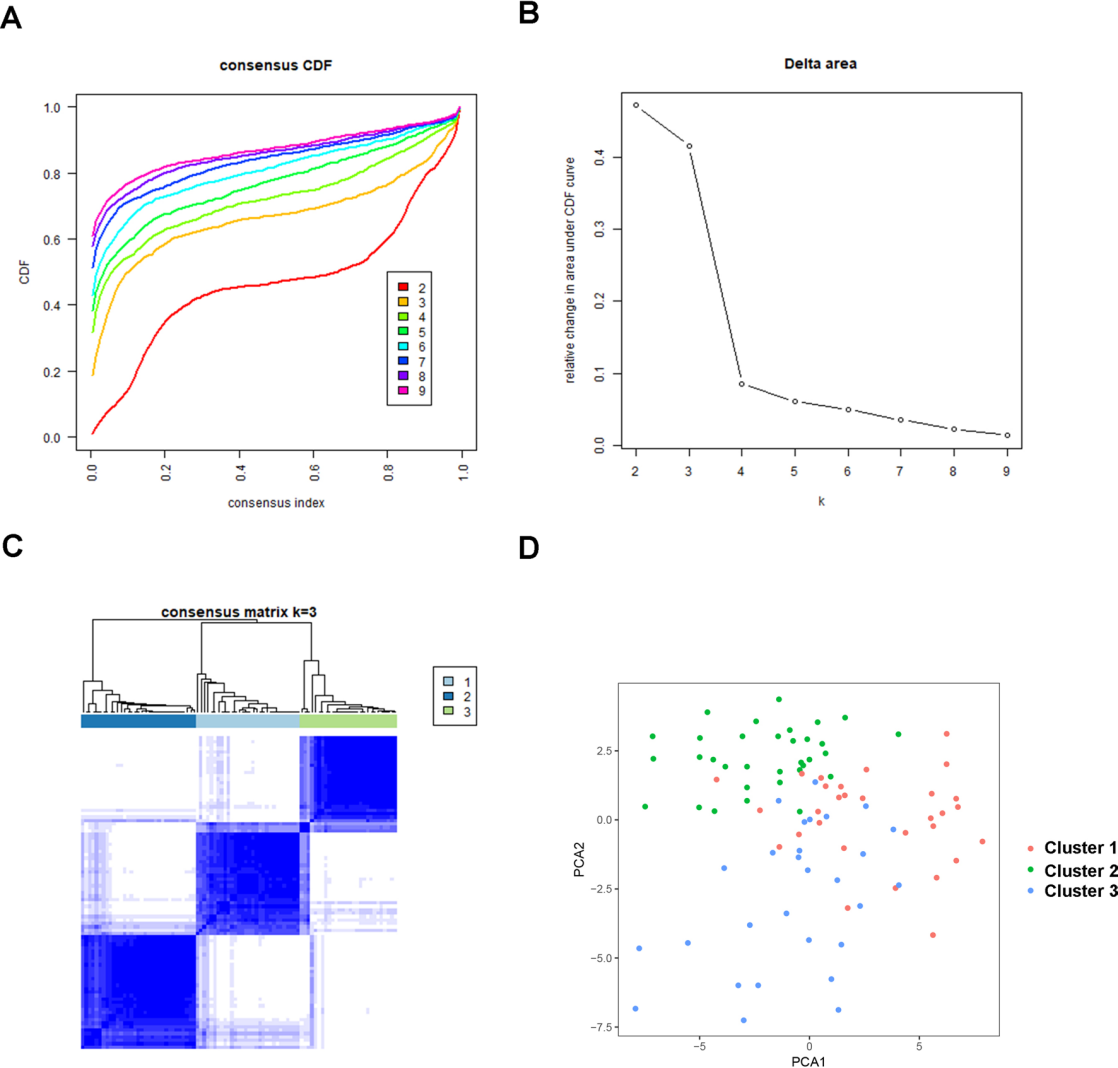
Consensus clustering for the ccRCC immune genes and immune related lncRNA expression in the ICGC dataset. (**A**) Consensus clustering cumulative distribution function (CDF) for k = 2 to 9. (**B**) The Relative change in area under CDF curve for k = 2 to 9. (**C**) Consensus matrix heatmap plots when k = 3. (**D**) Principal component analysis of the immune genes and lncRNAs expression when k = 3.

According to the expression profile of immune genes and immune-related lncRNAs, we employed a consensus clustering algorithm to categorize the ccRCC patients into de novo groups. As indicated in Fig. 7A,B, we performed a consensus clustering algorithm via the Cancersubtype R package and revealed that the area under the cumulative distribution function (CDF) curve maintained a maximal consensus within clusters and a minimal ambiguity rate in cluster assignments when k = 3. Therefore, the dataset was finally partitioned into three groups: cluster 1 (N = 182), cluster 2 (N = 179), and cluster 3 (N = 152) (Fig. 7C). Then, the Principal Component Analysis (PCA) analysis was performed on the expression of immune genes and immune-related lncRNAs on the three clusters. A clear distinction across the three clusters was noted (Fig. 7D). survival analysis showed that cluster 2 had a better survival time compared to cluster 1 and cluster 3 (Fig. 8A). The three clusters with expression data and clinical traits were presented in Fig. 8B. Besides, a consensus clustering algorithm on the ICGC dataset was used to validate the feasibility and reliability of the three molecular subtypes. The area under the cumulative distribution function (CDF) curve was calculated to determine the optimal k value (Fig. 9). Notably, after a comprehensive consideration, k = 3 was selected as the optimal cluster number. Furthermore, PCA results indicated divergence in the three subgroups. However, due to the small sample size (N = 91), the survival outcome (*p* = 0.051) in the subgroups was insignificant (Supplementary Fig. 6 ).

### Identification of Immunogenomic profiling

**Figure 10:**
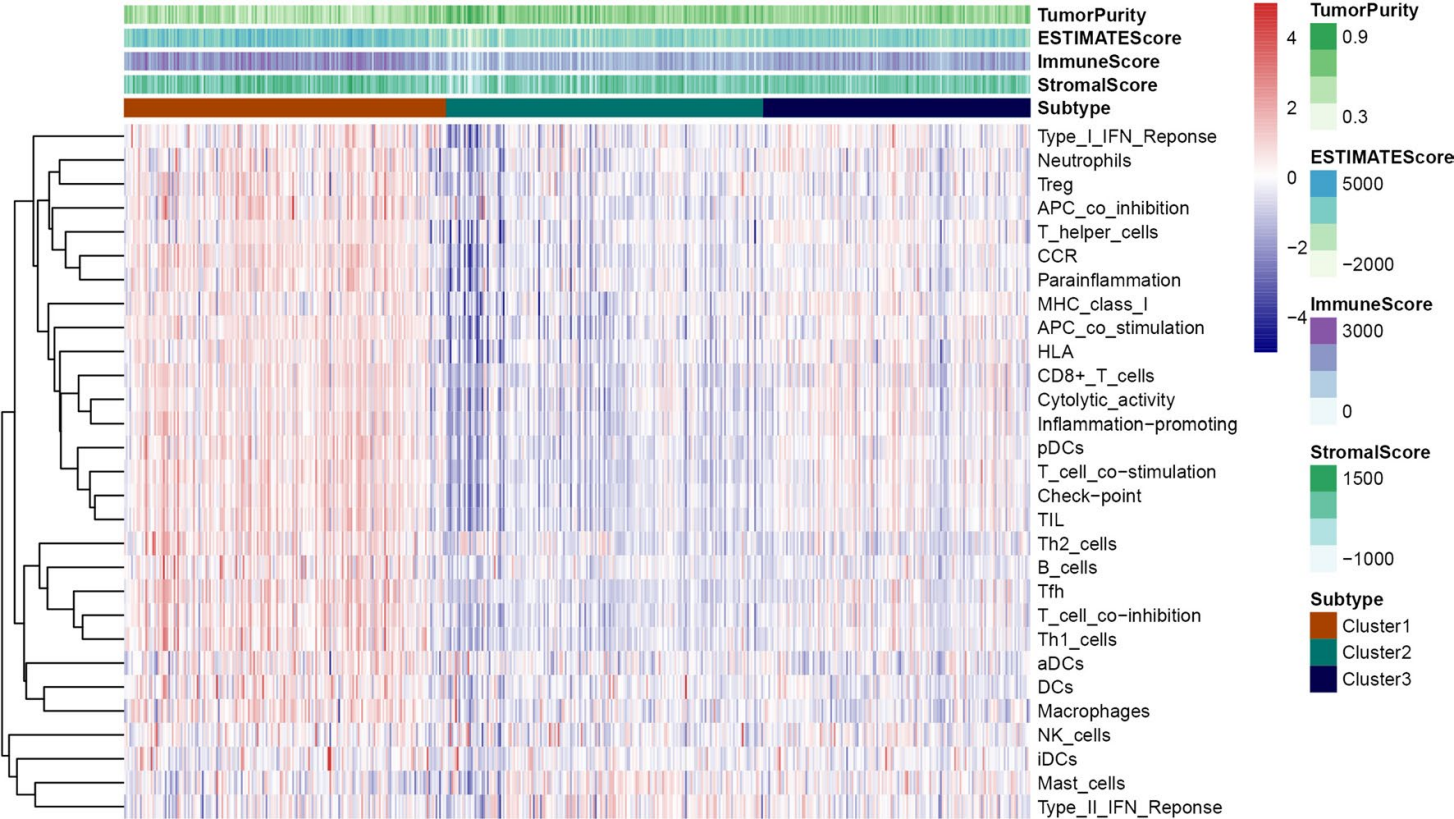
Hetamap of the immunity ordered by the three subtypes. The cluster 2 corresponding to low immunity, cluster 1 corresponding to high immunity and cluster 3 associated with median immunity. The Tumor_purity, Stromal_score, and Immune_score were estimated by ESTIMATE.

**Figure 11:**
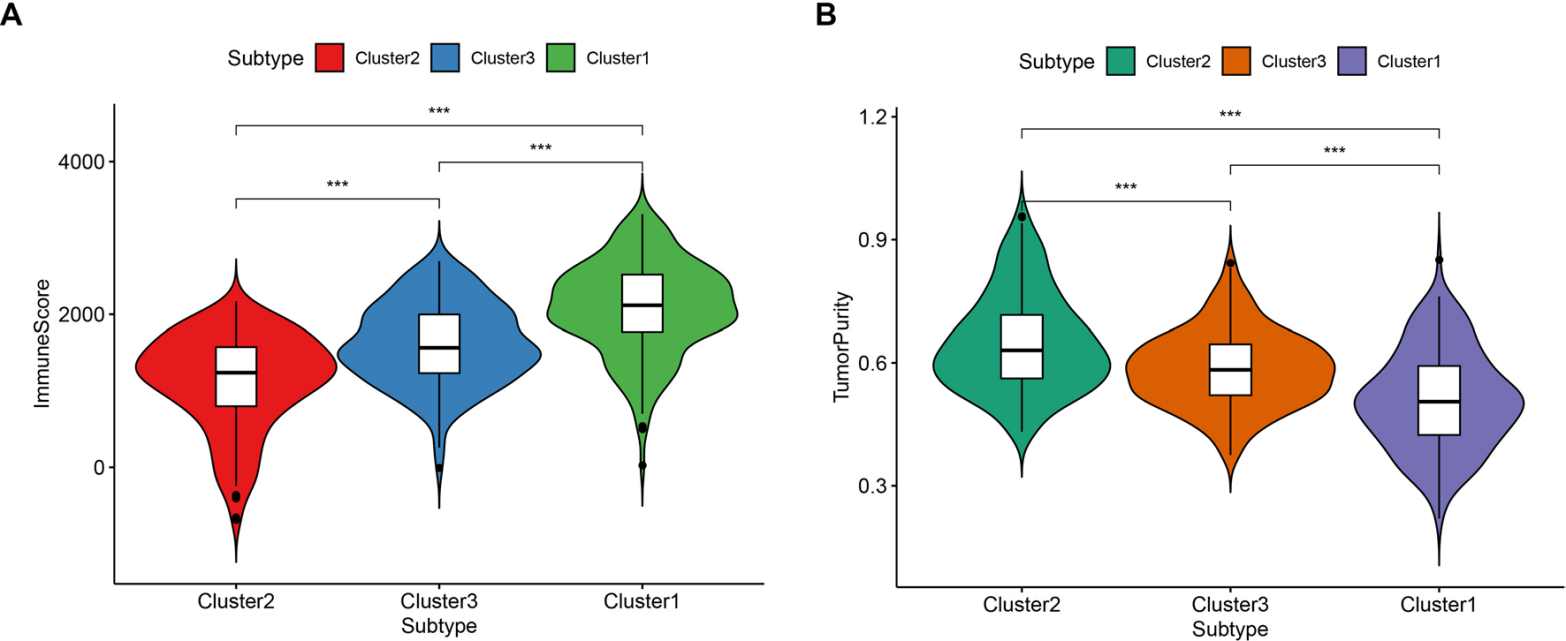
Violin plot for the immune score (**A**) and tumor purity (**B**) in different subtypes.

The 29 immune-related gene sets represented immune cell types, functions, and pathways that were fitted into the analysis (Supplementary Table 3). Of note, the ssGSEA score was used to quantify enrichment levels of the immune cells, function, and pathways in ccRCC samples. The immune scores were further classified into three clusters corresponding to the previously partitioned cluster groups. Interestingly, the high immune infiltration level corresponded to cluster 1, median immune infiltration level corresponded to cluster 3 whereas, low immune infiltration level corresponded to cluster 2 (Fig. 10). Also, the immune score and tumor purity were analyzed using the ESTIMATE R package. The immune scores were significantly higher in cluster 1 while the scores were significantly lower in cluster 2 (Kruskal–Wallis test, *P* < 0.001) (Fig. 11A). However, we noted a reverse trend on tumor purity which was significantly higher in cluster 2 but significantly lower in cluster 1 (Kruskal–Wallis test, *P* < 0.001) (Fig. 11B). Generally, these findings indicated that the cluster was highly associated with immunity. Of note, cluster 2 comprised more tumor cells whereas, cluster 1 had more immune cells.

## Discussion

Unlike protein-coding genes, lncRNAs are involved in many important biological functions linked to human disease, epigenetic and post-transcription regulation^17^. Current literature shows that the non-coding RNAs, specifically lncRNAs, are tightly associated with tumorigenesis and progression of tumors. Also, cumulative evidence reveals that dysregulated lncRNAs may serve as a critical biomarker for many types of cancers, in particular, esophageal squamous cell carcinoma and breast cancer^18,19^. Numerical evidence pronounce that lncRNAs can associate with miRNA to weaken the effects of miRNA on in mediating mRNA expression^20^. Some recent studies have suggested that aberrant lncRNA expression is associated with ccRCC, and many potential biomarkers have been uncovered in ccRCC^21–23^. However, these results rely on a temporary TCGA database that lacks external validation sets, hence, it poses difficulty in assessing the accuracy of the model. Moreover, due to the complexity of ccRCC, there is an urgent need to identify a large number of lncRNA markers for the prognosis and diagnosis of ccRCC. Presently, immunotherapy is a commonly used tumor intervention. However, for tumors and ccRCC, it exhibits a remarkable immune infiltration and other immune-related signatures^24^. Nevertheless, an increasing number of immunotherapy compounds such as PD-1/PD-L1 blocking agents have been approved in the treatment of ccRCC and have achieved noticeable progress^25^. However, few patients are unresponsive due to tumor heterogeneity, and cases of drug resistance occur^6^. Therefore, individualized immunotherapy treatment for patients is highly preferable.

In the present study, we included four GEO datasets (GSE46699, GSE36895, GSE15641, and GSE53757). Exactly 49 immune genes associated with ccRCC were screened from the GEO datasets using RRA methods. Through correlation analysis, 140 high confidence lncRNAs were identified in the TCGA dataset. Furthermore, a 12 immune-related lncRNA signature was developed and validated. This was performed through univariate cox regression and LASSO-penalized multivariate Cox proportional hazards modeling analyses in the TCGA and ICGC dataset. K–M curves analysis identified a significant divergence in patients classified in the high-risk group and low-risk group. Besides, GSEA enrichment analysis demonstrated that low-risk group patients exhibit significantly enriched pathways such as the ERBB signaling pathway, MAPK signaling pathway, pathways of cancer, renal cell carcinoma, TCG beta signaling pathway, and WNT signaling pathway. Thus, we suggest that the 12-lncRNA prognostic signature plays a crucial role in molecular pathogenesis, progression, and prognosis of ccRCC. Moreover, we compared our 12-lncRNA signature with clinical traits (Age, Gender, Stage, Grade, Smoking, T, N, and M) through ROC analysis, whereby, we demonstrated that our risk model is optimal (AUC = 0.783). Using cox regression analysis (p < 0.001), it was revealed that the 12-lncRNA signature can independently predict clinical traits. Therefore, these results imply that the 12-lncRNA prognostic signature may provide a reliable prognostic marker and a theoretical basis on the mechanism of ccRCC. To explore the potential molecular subtype of ccRCC, we exploited the immune gene expression profile. We further identified three subtypes (cluster 1, cluster 2, and cluster 3) that are linked to the overall survival of ccRCC. Compared to cluster 1 and cluster 3, Log-rank test suggested that cluster 2 exhibited a better survival outcome (p < 0.05). Interestingly, we observed that cluster 1 displayed a high immune infiltration level whereas cluster 2 showed a low immune infiltration level. Notably, cluster 3 correlated with the median immune infiltration level. Recent studies identified similar subgroup results through the ssGSEA methods based on the immune gene sets^27^. In addition, 3 lncRNAs including, AC005104.1, AL360181.2, and LINC01011) showed a significantly high expression level in cluster 1 when compared to cluster 2. This is an indication that the 3 lncRNAs are positively correlated with immunity (Supplementary Fig. 7).

We further identified 12 prognostic associated lncRNAs in ccRCC. including AC005104.1, AC093278.2, AC098484.1, AL360181.2, LINC01011, AP001372.2, AC007637.1, AL354733.3, and AP001189.3, which were the first time that served as markers in the current research. The lncRNA EMX2OS, which has been validated and identified to potentially induce proliferation and invasion, and promote sphere formation in ovarian cancer cells by regulating the miR-654-3p/AKT3/PD-L1 Axis^28^ Additionally, EMX2OS can be used as a biomarker in laryngeal cancer and papillary thyroid cancer^29,30^. Furthermore, SPINT1-AS1 was identified as a potential marker in the prognosis of colorectal cancer. High expression levels of SPINT1-AS1 was reported to correspond with a worse survival outcome^31^. There has been a high expectation on LINC00886 as a novel biomarker in laryngeal carcinoma^32^. However, the lncRNA which is a potential biomarker should be subjected to further experimental validation in future studies.

In conclusion, we identified 12 immune-related lncRNAs that correlated with the survival of ccRCC. Further, we discovered three molecular subtypes that are associated with tumor immunity. Our study has important clinical significance in understanding the underlying mechanism of ccRCC, therefore, can be used as a reference by clinicians for individualized treatment.

## Methods

### Data collection

The four microarray datasets (GSE46699, GSE36895, GSE15641, and GSE53757) were downloaded from the GEO database (www.ncbi.nlm.nih.gov/geo) as per the keyword ‘renal cell carcinoma’. Notably, the GPL570 version platform was adopted to obtain datasets (GSE46699, GSE36895, and GSE53757). Dataset GSE46699 comprised 63 adjacent normal kidney specimens and 67 ccRCC samples whereas, the GSE36895 dataset comprised 23 adjacent normal kidney samples and 29 ccRCC samples. Dataset GSE53757 composed of 72 adjacent normal kidney samples and 72 ccRCC samples. The platform used to obtain the GSE15641 dataset was based on the GPL96 version which incorporated 23 normal kidney samples and 32 ccRCC samples. All the expression level data were standardized and log2 transformed. In addition, 539 and 91 ccRCC samples were downloaded with corresponding clinical information from the TCGA database and ICGC database respectively.

### Integration of microarray data

The differentially expressed genes (DEGs) were identified for the four datasets through Limma analysis^11^. Gene expression analysis of the four microarray datasets was conducted using the RRA R package. A comparison of the expression level of genes was conducted according to their ranks. Screening of the significantly expressed genes were based on the p-adjusted < 0.05. Besides, the immune-related genes were downloaded from the IMMPORT database (https://www.immport.org/) and the significantly immune genes in our datasetwere obtained from the RRA result^12^.

### Correlation analysis

Data on the immune genes and lncRNAs expression of ccRCC were extracted from the TCGA dataset. The correlation of lncRNAs with immune genes was determined according to the Pearson coefficient, whereby, we performed cor function using the R package. The immune-related lncRNAs were identified with the criterion: Pearson coefficient > 0.5 and p-value < 0.0001.

### Identification and validation of lncRNA prognostic signature

To perform survival analysis, we integrated the survival time and the corresponding lncRNA expression. The ccRCC patient samples with immune-related lncRNAs were equally categorized into training and testing datasets. Further, through univariate Cox regression analysis, we selected significantly correlated lncRNAs. For a robust and reliable lncRNA, significantly correlated lncRNA with p-value < 0.05 were selected and fitted into Lasso-penalized multivariate Cox proportional hazards modeling analysis. After 1,000 iterations using the LASSO analysis, the lncRNAs with frequency > 50 were selected and fitted into multivariate cox regression analysis. The risk score formula was established based on the regression coefficients as follows: Risk score = ∑Coef_lncRNAs_ × Exp_lncRNAs_. The Coef_lncRNA_ denoted the lncRNA regression coefficient, where Exp _lncRNA_ denoted the expression level of the corresponding lncRNA. Thereafter, the risk score for each patient was calculated and subsequently categorized into high-risk group and low-risk group based on the median risk score in the training and testing datasets, respectively. Kaplan–Meier curve analysis was conducted via the R survival package (https://cran.r-project.org/web/packages/survival) to estimate the survival divergence between high-risk and low-risk groups. ROC curve analysis was aided in evaluating the accuracy of the prognostic model via the survival ROC package (https://cran.r-project.org/web/packages/survivalROC). Also, the lncRNA prognostic signature was validated in the ICGA dataset. The risk score for each patient in the ICGC dataset was calculated according to the risk model. The survival difference between risk groups was implemented using K–M curve analysis.

### GSEA enrichment analysis

Gene set enrichment analysis was performed between the high-risk group and low-risk group. The significant pathways were enriched with the NOM p-value < 0.05 and FDR < 0.25. The c2.cp.kegg.v7.0.symbols.gmt was selected as the reference file.

### Molecular subtype identification and validation

The potential molecular subtypes were identified using the “ExecuteCC” function from the Cancersubtypes R package"^13^ based on the expression of immune genes and lncRNAs. Principal components analysis (PCA) was performed to validate the subtypes of ccRCC. Further, the survival rate of different subtypes was conducted using K–M curve analysis. The molecular subtypes were further validated in the ICGA dataset based on the expression of immune lncRNA and gene expression using “Cancersubtypes” R package.

### Gene signature obtained and ssGSEA analysis

The marker genes for immune cell types were reffered from a previous study by Bindea et al*.*^14^. Infiltration levels for each of the immune cell types were estimated by the ssGSEA method from GSVA R package^15^. Then, we transformed each attribute (immune signature or gene set) value (ssGSEA score) xi into *xi′* by the equation *xi′* = (xi − xmin)/(xmax − xmin), where,. xmin and xmax denoted the minimum and maximum of the ssGSEA scores for the gene set across all ccRCC samples, respectively.

### Evaluation of the immune score, tumor purity, and stromal score in ccRCC

ESTIMATE algorithm was used to calculating the fraction of immune and stromal cells in tumor tissues based on a gene expression signature. The R script of the ESTIMATE algorithm was downloaded from the public source website (https://sourceforge.net/projects/estimateproject/). This was followed by a calculation of the Immune scores, stromal scores, and ESTIMATE scores for each ccRCC patient^16^.

### Statistical analysis

All the computational and statistical analyses were conducted on the R software (version3.6.2). Kruskal–Wallis test was used to compare the divergence between multiple groups. Chi-square test or Fisher exact test was used for statistics on clinical information. A Bonferroni test was used to correct the p-value. Kaplan–Meier curves analysis was used to assess survival differences of subtype.

## Supplementary Information 1.

Supplementary information.

## Data Availability

The dataset performed in this study are available from the corresponding author on reasonable requests.
