# Supplementary material for: Identification of 12 immune-related lncRNAs and molecular subtypes for the clear cell renal cell carcinoma based on RNA sequencing data
Source: Sci Rep. 2020 Sep 2;10:14412. doi: 10.1038/s41598-020-71150-3 (PMC7467926; doi:10.1038/s41598-020-71150-3)
Supplement: Supplementary file 1 — Supplementary information. [file 41598_2020_71150_MOESM1_ESM.pdf]

Identification of 12 immune-related lncRNAs and molecular subtypes for the clear cell renal cell carcinoma based on RNA sequencing data

Weimin Zhong[1#], Bin Chen[4#], Hongbin Zhong[1], Chaoqun Huang[1], Jianqiong Lin[1], Maoshu Zhu[1], Miaoxuan Chen[1], Ying Lin[1], Yao Lin[2*], Jiyi Huang[1, 3*]

Supplementary information

Supplementary Figure 1

A logFC heatmap for each GEO datasets (GSE46699, GSE36895, GSE15642 and GSE53757), the red label represent up-regulated while the green label represent down-regulated.

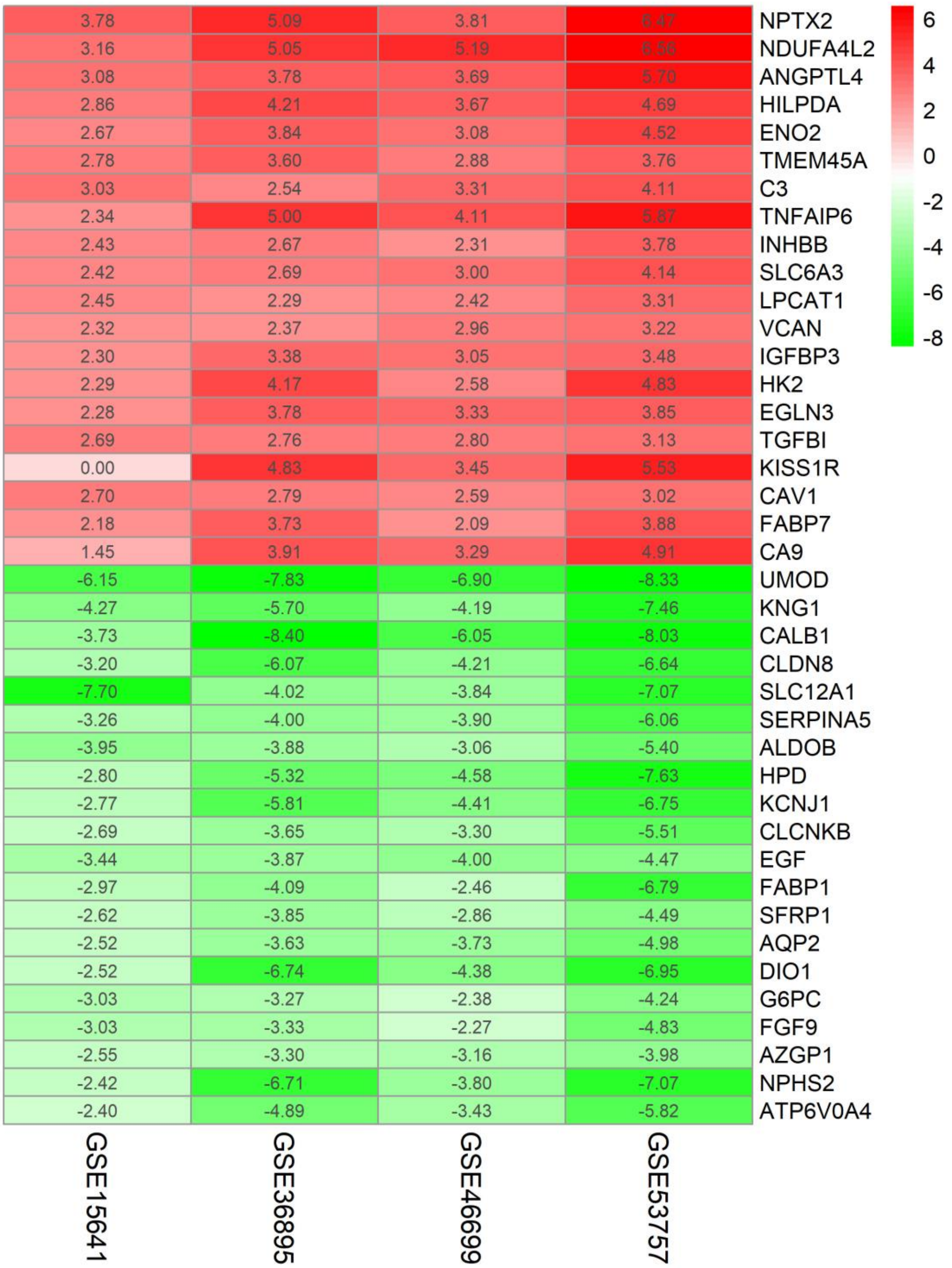

Supplementary Figure 2

KM curve survival analysis for the risk groups in the ICGC dataset

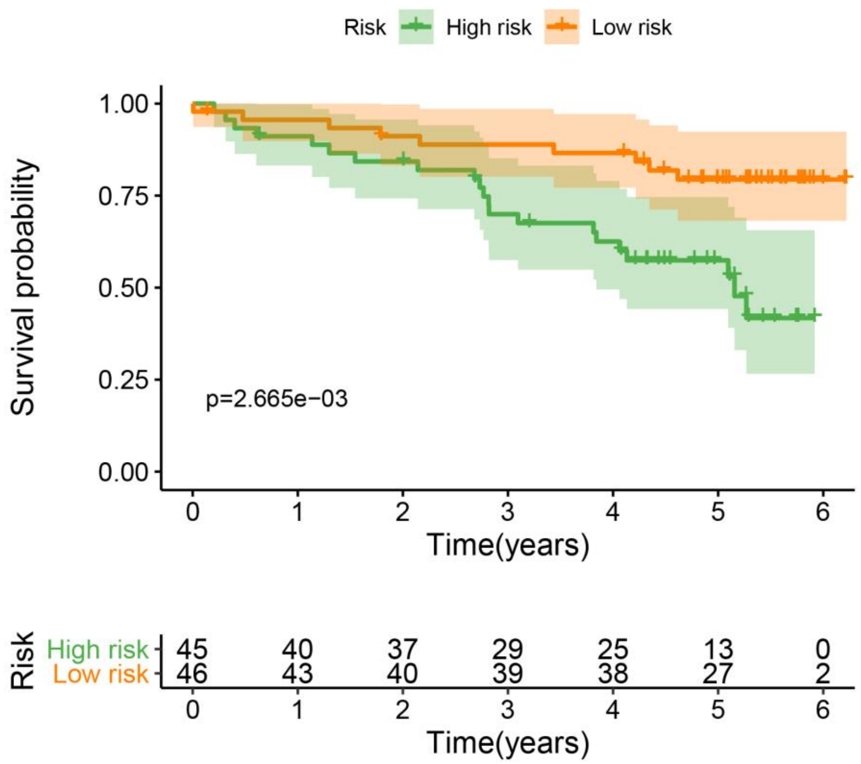

Supplementary Figure 3

Time ROC curves analysis for the 12-lncRNA signature in the ICGC dataset

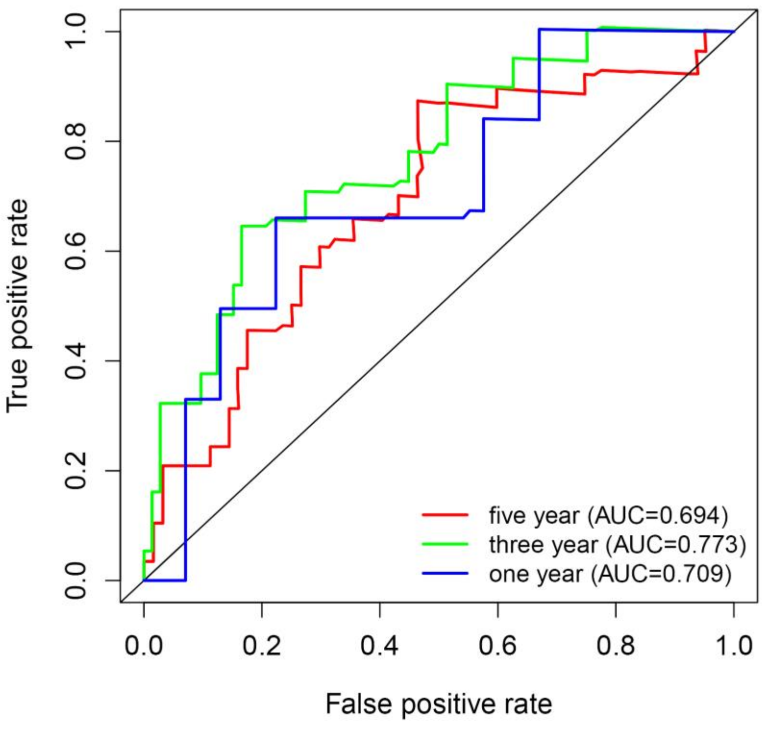

Supplementary Figure 4

Roc curve analysis for the 12-lncRNA signature and clinical traits (Stage, Gender, Age, Grade, T, M, N and Smoking)

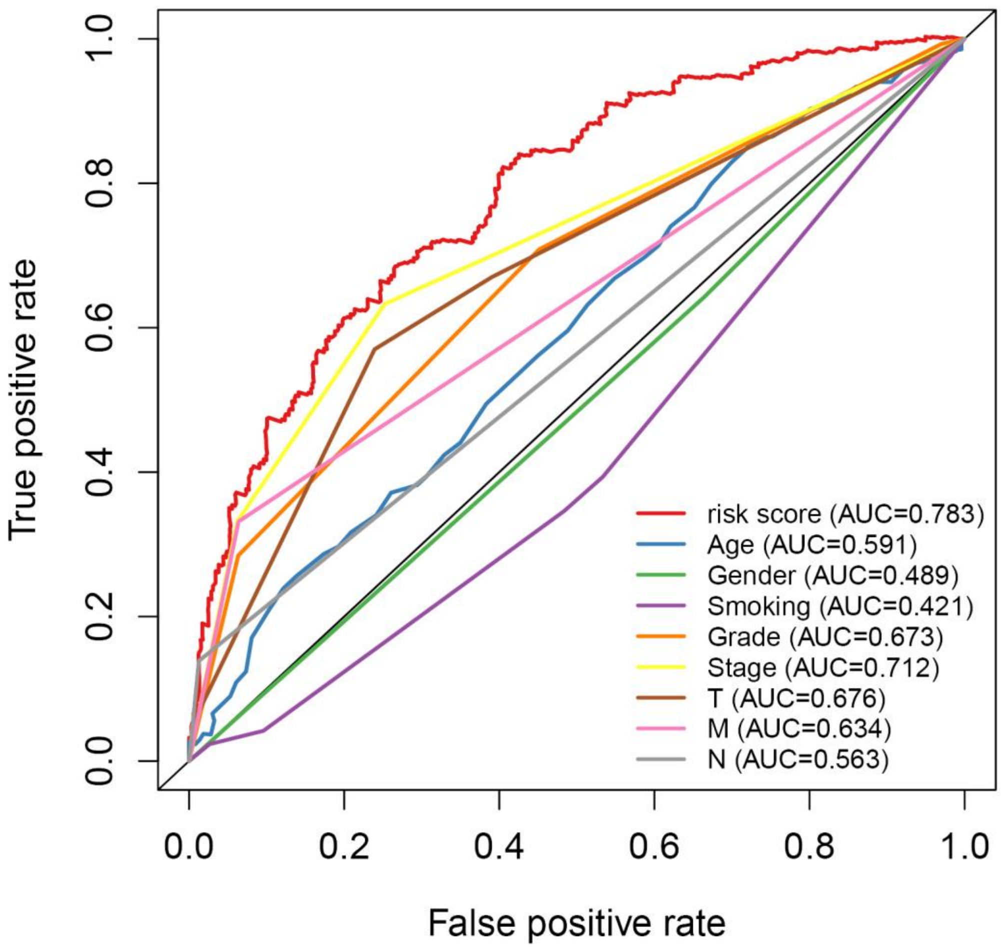

Supplementary Figure 5

The association between 12 lncRNAs expression and clinical features. (A) Boxplots of 12 lncRNAs from AC005104.1 to SPINT1-AS1 in different pathological stages. (B) Botplots for 12 lncRNAs from AC005104.1 to SPINT1-AS1 in different grade. ns: no significant; *P < 0.05; **P < 0.01; ***P < 0.001.

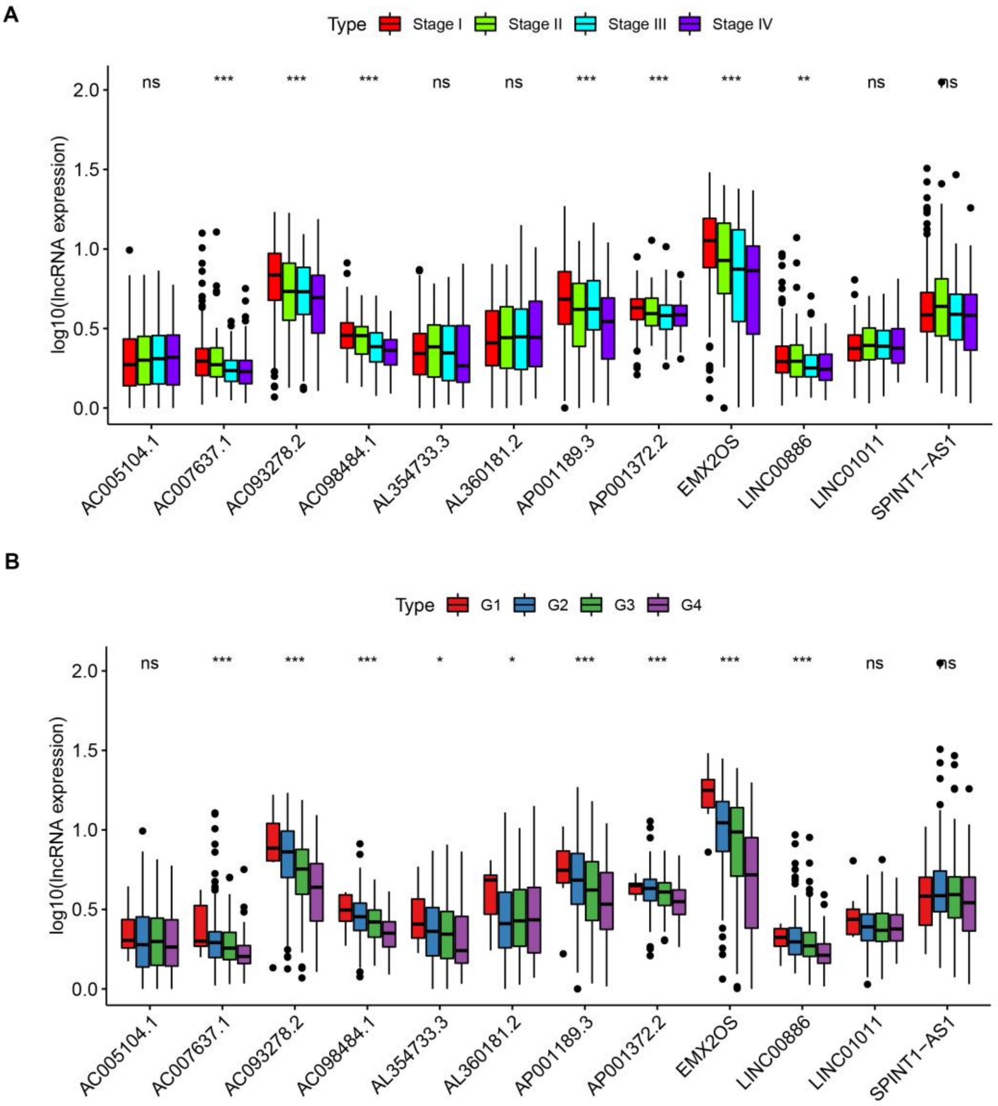

A
Type Stage I Stage II Stage III Stage IV
log10(lncRNA expression)
ns *** *** *** ns ns *** *** *** ** ns ns
AC005104.1
AC007637.1
AC093278.2
AC098484.1
AL354733.3
AL360181.2
AP001189.3
AP001372.2
EMX2OS
LINC00886
LINC01011
SPINT1-AS1
B
Type G1 G2 G3 G4
log10(lncRNA expression)
ns *** *** *** * * *** *** *** *** ns ns
AC005104.1
AC007637.1
AC093278.2
AC098484.1
AL354733.3
AL360181.2
AP001189.3
AP001372.2
EMX2OS
LINC00886
LINC01011
SPINT1-AS1

Supplementary Figure 6

KM curves analysis for the overall survival of different subtypes in the ICGC dataset.

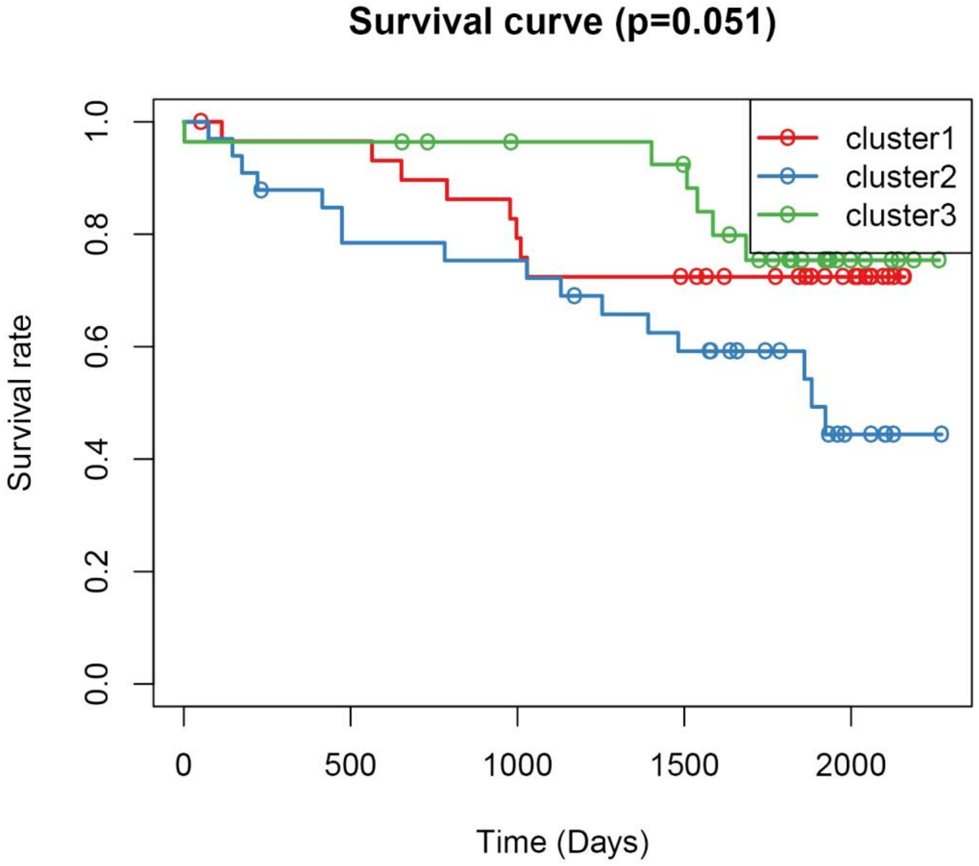

Supplementary Figure 7

Estimation of the relationships between 12 lncRNAs and three subgoups

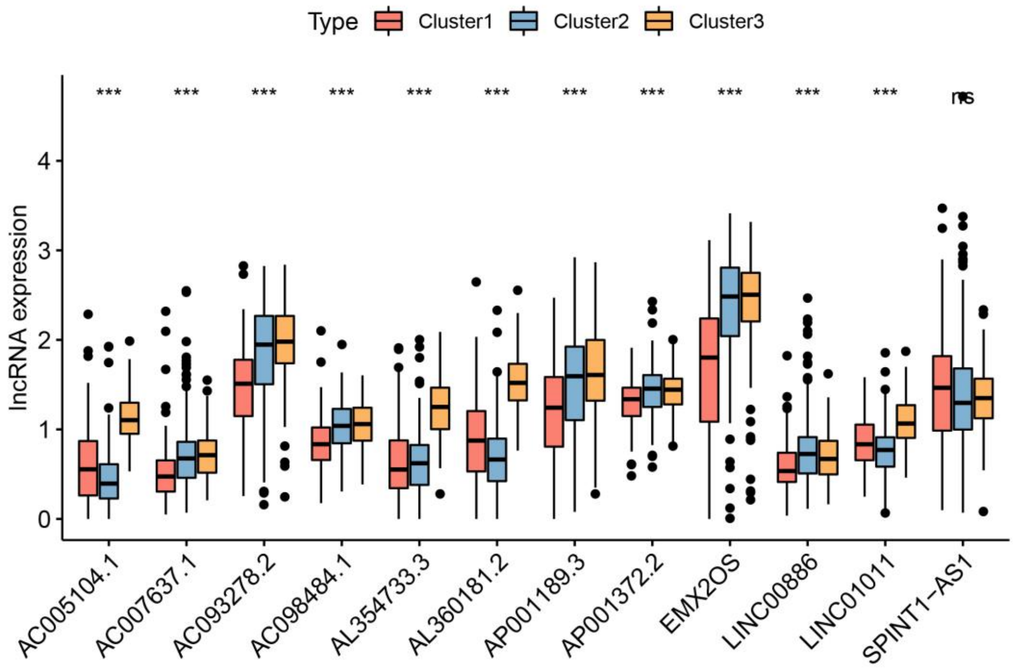

Supplementary Table 1

326 DEGs information that identified by RRA analysis

Name	Pvalue	adjPvalue	logFC
NPTX2	1.47E-15	3.18E-11	4.7863709
NDUFA4L2	2.65E-13	5.75E-09	4.9919465
ANGPTL4	6.96E-13	1.51E-08	4.0622925
HILPDA	2.36E-12	5.12E-08	3.8568892
ENO2	1.90E-11	4.12E-07	3.5284086
TMEM45A	2.72E-11	5.89E-07	3.255033
C3	3.78E-11	8.19E-07	3.2484773
TNFAIP6	4.24E-11	9.19E-07	4.3315033
INHBB	8.11E-11	1.76E-06	2.79854
SLC6A3	9.61E-11	2.08E-06	3.0634197
LPCAT1	1.78E-10	3.86E-06	2.6174957
VCAN	2.19E-10	4.76E-06	2.7164757
IGFBP3	2.19E-10	4.76E-06	3.05388
HK2	2.51E-10	5.44E-06	3.4673883
EGLN3	2.68E-10	5.80E-06	3.3108516
TGFBI	2.85E-10	6.18E-06	2.8444058
KISS1R	3.39E-10	7.35E-06	3.4525328
CAV1	4.87E-10	1.06E-05	2.7769282

FABP7	4.87E-10	1.06E-05	2.9700474
CA9	1.57E-09	3.40E-05	3.3905242
NETO2	1.81E-09	3.93E-05	2.9325383
CXCR4	2.85E-09	6.18E-05	2.5036095
C1QA	4.14E-09	8.99E-05	2.2734821
C1QB	5.50E-09	0.0001192	2.8466761
BIRC3	5.50E-09	0.0001192	2.279771
PLK2	5.50E-09	0.0001192	2.3617024
FABP5	6.38E-09	0.0001383	2.5769971
LYZ	8.01E-09	0.0001735	2.2242749
LOX	8.23E-09	0.0001784	2.770614
DDIT4	8.69E-09	0.0001884	2.1676884
NOL3	1.05E-08	0.0002266	2.0848482
APOC1	1.19E-08	0.0002573	2.4643647
AHNAK2	1.31E-08	0.000284	2.2726854
CP	1.45E-08	0.0003151	3.1804336
RGS1	1.48E-08	0.0003202	2.4821054
COL23A1	1.67E-08	0.0003622	2.6559239
CCL5	1.99E-08	0.0004308	2.0112918
CTHRC1	2.17E-08	0.0004702	2.5537755
BHLHE41	2.21E-08	0.0004801	2.828556
GZMA	2.57E-08	0.0005561	1.9674084
VWF	2.84E-08	0.0006157	2.3180794
ZNF395	2.90E-08	0.0006281	1.9513641
SPAG4	3.08E-08	0.0006667	2.8560118
TYROBP	3.20E-08	0.0006934	2.3634499
CYP2J2	3.20E-08	0.0006934	2.4626315
CCND1	3.39E-08	0.0007348	2.0313847
STC2	3.39E-08	0.0007348	1.8960054
LAPTM5	3.59E-08	0.0007782	2.151237
C10orf10	3.66E-08	0.000793	1.9924676
ADM	4.09E-08	0.0008867	1.9548955
PLOD2	4.17E-08	0.0009031	2.2480821
OLFML2B	4.98E-08	0.0010797	1.8649286
SCARB1	5.62E-08	0.0012178	1.9244853
PSMB9	6.42E-08	0.0013916	2.1166646
TLR3	8.02E-08	0.0017395	1.7345806
GAL3ST1	8.02E-08	0.0017395	1.9932018
CAV2	8.28E-08	0.0017941	1.8230404
LGALS1	9.62E-08	0.0020864	2.0593966
ITGB2	1.05E-07	0.0022781	1.8625685
DDB2	1.10E-07	0.0023788	1.8376203
DIRAS2	1.26E-07	0.0027386	2.5417737
BTN3A2	1.28E-07	0.0027767	1.7420793

CD163	1.30E-07	0.0028152	1.7298787
CSTA	1.43E-07	0.003096	2.0815353
TAP1	1.63E-07	0.003533	1.8190601
EVI2A	1.67E-07	0.0036257	1.798937
NKG7	1.74E-07	0.0037681	2.2841049
APOLD1	1.81E-07	0.0039146	1.9802688
TIMP1	1.85E-07	0.0040147	1.9175166
ANGPT2	1.85E-07	0.0040147	2.5214989
VEGFA	1.92E-07	0.0041683	1.8761981
SCD	1.97E-07	0.0042732	1.6654676
GPR65	2.15E-07	0.0046558	1.9243817
CCL20	2.15E-07	0.0046558	2.1890913
CCL18	2.23E-07	0.0048275	1.9241498
KCNE4	2.57E-07	0.0055618	2.1913205
IL10RA	2.81E-07	0.0060959	1.6457563
SAP30	2.84E-07	0.0061653	1.5678086
MCAM	2.88E-07	0.0062353	1.8886028
PLA2G7	2.91E-07	0.0063058	1.9797028
ERV3-2	2.97E-07	0.0064487	1.7474621
FCGR1B	3.39E-07	0.0073578	1.8572043
HCLS1	3.43E-07	0.0074377	1.9091474
ACKR3	3.62E-07	0.0078468	1.8591226
GJA1	3.74E-07	0.0081003	1.7909806
CORO1A	3.74E-07	0.0081003	1.7692655
LY96	3.90E-07	0.0084477	1.8968525
FAM57A	3.94E-07	0.0085363	1.6928494
HEY1	4.10E-07	0.0088975	1.623948
PSMB8	4.28E-07	0.0092701	1.6735749
FCER1G	4.87E-07	0.0105624	1.9565
PRKCDBP	4.97E-07	0.0107726	1.7288102
IFI16	5.07E-07	0.0109859	1.8654652
C1S	5.58E-07	0.0121004	1.5066805
RNASE6	6.31E-07	0.0136734	1.652508
LHFPL2	6.73E-07	0.0145814	1.5305985
ELTD1	7.04E-07	0.0152572	1.587028
KCNK3	7.17E-07	0.0155339	1.5182363
FLT1	7.43E-07	0.0160988	1.4132457
TYMP	7.49E-07	0.0162423	1.6049008
RNASET2	7.90E-07	0.0171241	2.0379566
PNMA2	7.90E-07	0.0171241	1.6531169
ENPP3	8.63E-07	0.0187176	2.1397101
COL21A1	9.06E-07	0.0196511	1.6056756
MTCL1	9.14E-07	0.0198178	1.6452301
RRAD	9.22E-07	0.0199856	1.4394357

PTPRC	9.30E-07	0.0201544	1.4629682
SERPINE1	9.30E-07	0.0201544	1.4269578
PPP1R3C	9.30E-07	0.0201544	1.677792
FN1	9.77E-07	0.02119	1.7793841
CLEC2B	1.01E-06	0.0219022	1.5317692
EHD2	1.04E-06	0.022448	1.7242947
PCDH17	1.07E-06	0.0231916	1.6124668
VIM	1.10E-06	0.0237612	1.6384554
TRIB3	1.11E-06	0.0241468	1.8808469
KCNJ2	1.23E-06	0.0265597	1.7366367
CD70	1.28E-06	0.0278318	2.0567245
APOL1	1.30E-06	0.0282658	1.625524
RARRES2	1.39E-06	0.0300528	1.3879668
PYCARD	1.40E-06	0.030282	1.5895982
MS4A4A	1.42E-06	0.0307443	1.470912
DOC2A	1.45E-06	0.0314477	1.9833602
CHST15	1.46E-06	0.0316848	1.4663128
CXCL9	1.46E-06	0.0316848	1.7894825
COL6A2	1.53E-06	0.0331358	1.374133
ARL4C	1.54E-06	0.0333824	1.4111872
GZMB	1.60E-06	0.034636	1.9062136
LIPA	1.60E-06	0.034636	1.3966345
HCK	1.63E-06	0.035405	1.5470738
ITGA5	1.65E-06	0.0356641	1.5065179
TMCC1	1.69E-06	0.0367149	1.351145
ISG20	1.77E-06	0.0383345	1.6383164
ST8SIA4	1.78E-06	0.0386095	2.0824638
SERPINH1	1.82E-06	0.0394435	1.2929034
PRC1	1.93E-06	0.0417337	1.5385943
HLX	1.99E-06	0.0432148	1.4210671
COL1A2	2.04E-06	0.0441221	1.5866644
PECAM1	2.08E-06	0.0450437	1.3548869
ALOX5	2.09E-06	0.0453541	1.3737956
THEMIS2	2.12E-06	0.0459797	1.4483692
SLC2A3	2.21E-06	0.0478952	1.6811192
GMFG	2.25E-06	0.0488751	1.3596574
CD36	2.29E-06	0.0495367	1.7969086
SCG5	2.30E-06	0.04987	1.7758852
HLA-DMA	2.30E-06	0.04987	1.5056682
UMOD	2.90E-16	6.28E-12	-7.302804
KNG1	7.42E-14	1.61E-09	-5.404098
CALB1	3.76E-13	8.14E-09	-6.554177
CLDN8	2.15E-11	4.66E-07	-5.030439
SLC12A1	5.64E-11	1.22E-06	-5.658348

SERPINA5	6.19E-11	1.34E-06	-4.30207
ALDOB	8.84E-11	1.92E-06	-4.070806
HPD	1.66E-10	3.59E-06	-5.082931
KCNJ1	2.19E-10	4.76E-06	-4.935449
CLCNKB	2.68E-10	5.80E-06	-3.786769
EGF	3.65E-10	7.91E-06	-3.942262
FABP1	4.10E-10	8.90E-06	-4.07696
SFRP1	4.10E-10	8.90E-06	-3.456481
AQP2	7.05E-10	1.53E-05	-3.717898
DIO1	8.04E-10	1.74E-05	-5.144958
G6PC	9.45E-10	2.05E-05	-3.230212
FGF9	1.04E-09	2.25E-05	-3.362799
AZGP1	1.24E-09	2.69E-05	-3.247542
NPHS2	1.24E-09	2.69E-05	-4.998929
ATP6V0A4	1.41E-09	3.07E-05	-4.135511
TYRP1	1.48E-09	3.20E-05	-3.740351
SLC22A6	1.60E-09	3.48E-05	-3.507035
XPNPEP2	1.81E-09	3.93E-05	-4.047793
ALB	1.88E-09	4.09E-05	-3.460995
PCP4	1.96E-09	4.25E-05	-3.330601
HRG	2.04E-09	4.42E-05	-3.310422
DPEP1	2.12E-09	4.59E-05	-3.760058
PCK1	2.29E-09	4.96E-05	-3.756705
MAL	2.56E-09	5.54E-05	-3.692542
TSPAN8	2.85E-09	6.18E-05	-3.562915
ACSF2	3.76E-09	8.14E-05	-3.191834
TMEM213	4.31E-09	9.34E-05	-3.920067
HSD11B2	4.42E-09	9.58E-05	-3.581927
SOSTDC1	4.56E-09	9.90E-05	-3.21646
CYP4F3	4.71E-09	0.0001021	-3.428473
S100A2	5.33E-09	0.0001156	-3.495117
HAO2	5.50E-09	0.0001192	-2.910431
ABAT	6.20E-09	0.0001343	-2.887205
SLC12A3	7.16E-09	0.0001552	-3.254631
SLC13A1	7.79E-09	0.0001688	-3.152569
FXYD4	9.15E-09	0.0001984	-3.903004
DUSP9	1.16E-08	0.0002509	-2.894959
ALDH6A1	1.41E-08	0.0003054	-2.885324
SLC13A3	1.44E-08	0.0003127	-3.054985
DEFB1	1.58E-08	0.0003436	-3.405082
ASS1	1.90E-08	0.0004121	-2.758804
ESRRG	2.21E-08	0.0004801	-2.926568
TFAP2B	2.26E-08	0.0004904	-2.674896
TMEM52B	2.45E-08	0.0005315	-3.947865

DDC	2.51E-08	0.0005447	-2.414098
CYP4A11	2.57E-08	0.0005561	-2.513541
TUBB2B	3.08E-08	0.0006667	-2.530529
TCF21	3.14E-08	0.0006799	-2.496969
OGDHL	3.32E-08	0.0007208	-2.846837
AFM	4.24E-08	0.0009182	-3.60049
C7	4.72E-08	0.0010242	-2.750164
DCXR	4.98E-08	0.0010797	-2.585558
PLG	4.98E-08	0.0010797	-3.084024
PTH1R	5.25E-08	0.0011374	-2.973055
FAM151A	5.64E-08	0.001222	-3.310906
EFHD1	6.01E-08	0.0013025	-2.640471
SLC22A8	6.16E-08	0.0013365	-4.189292
LRRC19	6.42E-08	0.0013916	-2.532659
GLYAT	6.63E-08	0.0014377	-2.245246
FBP1	7.07E-08	0.0015336	-2.527832
PVALB	7.78E-08	0.0016862	-3.421883
PPP1R1A	9.48E-08	0.0020557	-2.412781
ATP6V1B1	9.77E-08	0.0021175	-2.401014
TOX3	9.91E-08	0.0021489	-2.266402
PCK2	1.23E-07	0.0026636	-2.338826
SCNN1G	1.59E-07	0.0034421	-2.743564
ALDH4A1	1.63E-07	0.003533	-2.364461
SORD	1.85E-07	0.0040147	-1.985296
IL17RB	1.88E-07	0.0040654	-2.02656
SLC34A1	1.90E-07	0.0041166	-2.611352
FTCD	1.97E-07	0.0042732	-1.89992
SLC5A12	1.97E-07	0.0042732	-2.366611
SLC7A9	2.00E-07	0.0043264	-2.008003
MT1F	2.07E-07	0.0044888	-2.189513
SLC27A2	2.07E-07	0.0044888	-2.08448
PEPD	2.28E-07	0.0049445	-2.03111
ATP6V1G3	2.33E-07	0.0050591	-3.239567
RALYL	2.47E-07	0.0053507	-3.660475
ACPP	2.51E-07	0.005434	-2.995437
CLIC5	2.51E-07	0.005434	-2.166733
AGMAT	2.57E-07	0.0055618	-2.172175
SOST	2.61E-07	0.0056533	-3.099937
LINC00645	2.75E-07	0.0059671	-3.193299
PLCL1	2.78E-07	0.0060272	-2.319684
MME	2.78E-07	0.0060272	-2.086422
CYP17A1	2.88E-07	0.0062353	-2.238106
SLC47A2	3.06E-07	0.0066291	-2.8782
FMO1	3.32E-07	0.0072	-2.055695

FAM169A	3.43E-07	0.0074377	-2.197552
PRODH2	3.43E-07	0.0074377	-2.270279
LPPR1	3.47E-07	0.0075182	-2.772715
DPYS	3.54E-07	0.0076812	-2.167162
UPP2	3.56E-07	0.0077111	-2.794842
AOX1	3.78E-07	0.0081861	-1.885688
GSTM3	3.98E-07	0.0086255	-2.163723
GATA3	3.98E-07	0.0086255	-2.330078
CTXN3	4.11E-07	0.0089047	-3.200629
PIPOX	4.15E-07	0.0089896	-2.78988
DCN	4.23E-07	0.0091759	-1.966154
KL	4.59E-07	0.0099502	-2.187749
CHL1	4.68E-07	0.0101513	-2.12606
CORO2B	5.17E-07	0.0112024	-1.781482
TMPRSS2	5.32E-07	0.011533	-2.242153
EPCAM	5.64E-07	0.0122163	-2.471577
CDH16	5.74E-07	0.0124507	-2.207442
CWH43	5.74E-07	0.0124507	-2.263498
KLK1	5.80E-07	0.0125691	-1.9413
MT1H	6.19E-07	0.013422	-2.070257
GGT6	6.35E-07	0.0137602	-2.706129
BHMT	6.66E-07	0.014449	-1.994456
MPPED2	6.85E-07	0.014849	-1.847225
HSPA2	7.10E-07	0.0153951	-2.19101
HS6ST2	7.15E-07	0.0155008	-2.950906
CAPN3	7.17E-07	0.0155339	-1.878915
HADH	7.23E-07	0.0156737	-1.913352
KCNJ15	7.36E-07	0.0159561	-1.960405
DAO	7.36E-07	0.0159561	-2.496216
MT1G	7.49E-07	0.0162423	-2.068359
GRB14	7.63E-07	0.0165324	-2.122586
SLC4A1	7.69E-07	0.0166788	-2.34437
HEPACAM2	7.72E-07	0.0167392	-3.24158
SLC44A4	8.04E-07	0.0174258	-1.810502
HMGCS2	8.39E-07	0.0181976	-1.951932
ANK2	8.61E-07	0.0186728	-1.772605
ERBB4	9.14E-07	0.0198178	-1.938282
FGF1	9.45E-07	0.0204953	-2.779317
ALDH8A1	9.61E-07	0.0208405	-2.166629
GPC5	9.62E-07	0.0208458	-3.226437
SCNN1A	9.94E-07	0.0215439	-2.229127
GK	1.01E-06	0.0219022	-1.575015
HYAL1	1.02E-06	0.022083	-1.568686
ATP6V0D2	1.03E-06	0.0223506	-2.771273

CYP24A1	1.04E-06	0.022448	-1.794136
NOX4	1.04E-06	0.0226322	-1.82919
CTH	1.06E-06	0.023004	-1.514472
MIOX	1.07E-06	0.0231295	-2.574922
CLDN10	1.08E-06	0.0233803	-1.809871
ARG2	1.10E-06	0.0239534	-1.880238
MST1L	1.11E-06	0.0241468	-1.840206
BPHL	1.12E-06	0.0243413	-1.70901
AKR7A3	1.13E-06	0.024537	-1.606421
MTTP	1.14E-06	0.0247339	-2.495126
MUC15	1.14E-06	0.0247409	-2.973386
AP1M2	1.16E-06	0.0251313	-1.695384
CYP4F2	1.18E-06	0.0255739	-3.209358
RGN	1.21E-06	0.0261455	-1.651565
NAP1L2	1.23E-06	0.0267686	-2.166378
ACOX2	1.23E-06	0.0267686	-1.953158
PDE1A	1.27E-06	0.0276167	-1.808258
EPHX2	1.30E-06	0.0282658	-1.689277
RBP4	1.30E-06	0.0282658	-1.806513
RP11-999E24.3	1.34E-06	0.0290926	-2.873693
RHCG	1.38E-06	0.03002	-3.038727
CRYAA	1.38E-06	0.03002	-3.063907
ACAA1	1.40E-06	0.030282	-1.762274
GPD1L	1.51E-06	0.0326467	-1.655589
CRYM	1.52E-06	0.0328906	-1.46971
RNF212B	1.56E-06	0.0339263	-2.604098
FOLR1	1.57E-06	0.0341304	-1.666048
TST	1.62E-06	0.0351473	-1.623348
AOC1	1.63E-06	0.035405	-1.717878
ABCB1	1.63E-06	0.035405	-1.779083
SELENBP1	1.74E-06	0.0377888	-1.525566
OLFM4	1.81E-06	0.0392671	-2.680395
PBLD	1.90E-06	0.041152	-1.906001
IGFBP2	1.90E-06	0.041152	-1.875242
SLC17A1	1.91E-06	0.0414421	-1.600745
ENPP6	1.92E-06	0.0415511	-3.150511
MT1E	1.97E-06	0.0426177	-1.763841
MT1X	2.05E-06	0.0444277	-1.662576
CRHBP	2.08E-06	0.0451402	-2.595646
RAB25	2.14E-06	0.0463809	-2.729915
ELF5	2.16E-06	0.0469301	-1.958689
DMRT2	2.20E-06	0.0476441	-2.454041
SLC7A13	2.26E-06	0.0489301	-3.273216
MAN1C1	2.27E-06	0.0492051	-2.063707

Supplementary Table 2

Correlation analysis for the immune related lncRNAs.

Immune Gene	lncRNA	cor	pvalue	Regulation
VEGFA	AL022328.1	0.540812527	2.86E-42	postive
TYMP	LINC01871	0.508138488	9.99E-37	postive
ISG20	LINC01871	0.567796453	2.50E-47	postive
CCL5	LINC01871	0.625785141	6.20E-60	postive
GZMB	LINC01871	0.558556219	1.52E-45	postive
FCER1G	LINC01871	0.514758382	8.41E-38	postive
VEGFA	AL662844.4	0.559137724	1.18E-45	postive
FGF9	AC104958.2	0.718882773	7.40E-87	postive
DEFB1	AC104958.2	0.509906312	5.19E-37	postive
FGF9	LINC01187	0.864982495	6.10E-163	postive
DEFB1	LINC01187	0.55521758	6.52E-45	postive
EGF	LINC01187	0.574224284	1.33E-48	postive
IL10RA	MIR155HG	0.534038466	4.53E-41	postive
CCL5	MIR155HG	0.6300528	5.73E-61	postive
FGF9	LINC01802	0.54634272	2.87E-43	postive
DEFB1	LINC01802	0.675600914	4.18E-73	postive
EGF	LINC01802	0.645974874	5.63E-65	postive
S100A2	HSD11B1-AS1	0.625938198	5.70E-60	postive
VEGFA	LINC00342	0.538603622	7.10E-42	postive
FGF9	HOXB-AS3	0.611727133	1.23E-56	postive
DEFB1	HOXB-AS3	0.761029298	5.35E-103	postive
FGF9	GATA2-AS1	0.61499606	2.18E-57	postive
DEFB1	GATA2-AS1	0.591568839	3.46E-52	postive
EGF	GATA2-AS1	0.540709987	2.99E-42	postive
FCER1G	AC098613.1	0.529475426	2.81E-40	postive
FLT1	AP001189.3	0.504994532	3.18E-36	postive
FGF9	AC007996.1	0.562322624	2.90E-46	postive
ITGB2	LINC01150	0.558921008	1.30E-45	postive
IL10RA	LINC01150	0.54321618	1.06E-42	postive
HCK	LINC01150	0.571305835	5.08E-48	postive
FCER1G	LINC01150	0.509767654	5.46E-37	postive
DEFB1	AC073896.2	0.60635435	2.03E-55	postive
FGF9	AC006547.1	0.542860526	1.23E-42	postive
VEGFA	AL928654.2	0.595133597	5.96E-53	postive
PSMB8	HCP5	0.565991586	5.64E-47	postive
HLA-DMA	HCP5	0.515694787	5.90E-38	postive
TAP1	HCP5	0.695368147	4.41E-79	postive
PTPRC	HCP5	0.500268625	1.77E-35	postive
C3	AC008760.2	0.540295727	3.54E-42	postive
DEFB1	LMO7-AS1	0.624484327	1.27E-59	postive

VEGFA	AL109811.2	0.53041949	1.93E-40	postive
VEGFA	AC138028.4	0.540369527	3.44E-42	postive
IL10RA	AC138207.5	0.584972121	8.47E-51	postive
DEFB1	ZNF503-AS2	0.564981395	8.87E-47	postive
EGF	LINC00645	0.528367494	4.36E-40	postive
ESRRG	LINC00645	0.588762525	1.36E-51	postive
GMFG	CYTOR	0.536299509	1.82E-41	postive
FLT1	AL158206.1	0.526850886	7.93E-40	postive
TYROBP	PCED1B-AS1	0.723328932	2.04E-88	postive
ITGB2	PCED1B-AS1	0.674308161	9.89E-73	postive
IL10RA	PCED1B-AS1	0.818681212	1.82E-131	postive
HLA-DMA	PCED1B-AS1	0.686623767	2.22E-76	postive
TYMP	PCED1B-AS1	0.604972766	4.13E-55	postive
HCK	PCED1B-AS1	0.604502202	5.26E-55	postive
ISG20	PCED1B-AS1	0.640811625	1.19E-63	postive
CCL5	PCED1B-AS1	0.855352412	1.66E-155	postive
TAP1	PCED1B-AS1	0.625646497	6.70E-60	postive
CXCL9	PCED1B-AS1	0.610462962	2.39E-56	postive
GZMB	PCED1B-AS1	0.554694558	8.17E-45	postive
PTPRC	PCED1B-AS1	0.623676067	1.99E-59	postive
FCER1G	PCED1B-AS1	0.771848621	1.08E-107	postive
BPHL	TMEM246-AS1	0.526202593	1.02E-39	postive
FGF9	AC148477.4	0.626775938	3.58E-60	postive
DEFB1	AC148477.4	0.68685065	1.90E-76	postive
EGF	AC148477.4	0.615255349	1.90E-57	postive
BPHL	AC007406.2	0.515145222	7.27E-38	postive
VEGFA	HIF1A-AS2	0.505335013	2.80E-36	postive
FGF9	AC055720.2	0.676763436	1.92E-73	postive
DEFB1	AC055720.2	0.540579851	3.15E-42	postive
EGF	AC055720.2	0.520802643	8.37E-39	postive
VEGFA	MIR210HG	0.53253073	8.31E-41	postive
VEGFA	AC009084.1	0.566663794	4.17E-47	postive
FGF9	AL606834.1	0.540407443	3.38E-42	postive
BPHL	U91328.1	0.584474111	1.07E-50	postive
VEGFA	LINC01011	0.520619403	8.98E-39	postive
VEGFA	AC090589.3	0.548938525	9.59E-44	postive
FGF9	AC103563.7	0.721660132	7.91E-88	postive
DEFB1	AC103563.7	0.764430941	1.91E-104	postive
EGF	AC103563.7	0.659924054	1.09E-68	postive
ESRRG	AC103563.7	0.508793474	7.84E-37	postive
FGF9	GAS6-DT	0.765216137	8.75E-105	postive
DEFB1	GAS6-DT	0.677166828	1.46E-73	postive
EGF	GAS6-DT	0.607369513	1.20E-55	postive
GMFG	AC011445.2	0.530957254	1.56E-40	postive

S100A2	AC011445.2	0.665272858	3.64E-70	postive
VEGFA	AC022150.2	0.532207947	9.45E-41	postive
TYROBP	LINC01857	0.658031122	3.58E-68	postive
CCL18	LINC01857	0.514030268	1.11E-37	postive
FCER1G	LINC01857	0.646355997	4.49E-65	postive
BPHL	LINC01507	0.599593713	6.39E-54	postive
GMFG	SNHG19	0.547160551	2.03E-43	postive
S100A2	SNHG19	0.701657801	4.36E-81	postive
VEGFA	AC007566.1	0.503019315	6.53E-36	postive
INHBB	AL355803.1	0.524946202	1.67E-39	postive
FLT1	AL355803.1	0.585184928	7.64E-51	postive
DEFB1	SPINT1-AS1	0.715536694	1.06E-85	postive
IL10RA	TNRC6C-AS1	0.538168869	8.48E-42	postive
FGF9	AC021087.4	0.535923045	2.11E-41	postive
DEFB1	AC021087.4	0.574416459	1.22E-48	postive
EGF	AC021087.4	0.54774615	1.59E-43	postive
GMFG	ZSCAN16-AS1	0.553604754	1.31E-44	postive
S100A2	ZSCAN16-AS1	0.675930202	3.35E-73	postive
FGF9	LINC00265	0.614765671	2.46E-57	postive
BPHL	ADAMTS9-AS1	0.514013752	1.11E-37	postive
FGF9	COLCA1	0.52458234	1.93E-39	postive
EGF	COLCA1	0.535129968	2.92E-41	postive
FGF9	LINC00886	0.628156417	1.66E-60	postive
DEFB1	LINC00886	0.580709745	6.43E-50	postive
EGF	LINC00886	0.625609074	6.84E-60	postive
ESRRG	LINC00886	0.615440225	1.72E-57	postive
FGF9	SCAMP1-AS1	0.652265441	1.26E-66	postive
DEFB1	SCAMP1-AS1	0.568383288	1.92E-47	postive
S100A2	AL513165.1	0.648513609	1.23E-65	postive
VEGFA	AL160006.1	0.573317568	2.02E-48	postive
DEFB1	CA3-AS1	0.595211499	5.73E-53	postive
TLR3	WDFY3-AS2	0.515238598	7.01E-38	postive
DEFB1	SEMA3B-AS1	0.6336219	7.59E-62	postive
BPHL	EMX2OS	0.546258211	2.97E-43	postive
IL10RA	HLA-DQB1-AS1	0.525487732	1.35E-39	postive
VEGFA	AL365330.1	0.573238823	2.09E-48	postive
INHBB	AC093278.2	0.51462083	8.86E-38	postive
FLT1	AC093278.2	0.768599698	2.96E-106	postive
S100A2	AC023509.3	0.551094959	3.83E-44	postive
IL10RA	ITGB2-AS1	0.519872398	1.20E-38	postive
TYMP	ITGB2-AS1	0.550389879	5.18E-44	postive
CCL5	ITGB2-AS1	0.517529542	2.94E-38	postive
FGF9	SBF2-AS1	0.687603491	1.12E-76	postive
DEFB1	SBF2-AS1	0.509121082	6.95E-37	postive

ESRRG	SBF2-AS1	0.519789609	1.24E-38	postive
VEGFA	HCG27	0.52153829	6.30E-39	postive
FGF9	AC007637.1	0.539132407	5.71E-42	postive
EGF	AC007637.1	0.507267713	1.38E-36	postive
VEGFA	AL360181.2	0.505071747	3.09E-36	postive
VEGFA	AP001830.1	0.538093199	8.74E-42	postive
FGF9	FGD5-AS1	0.504150883	4.32E-36	postive
DEFB1	AP000894.4	0.511855892	2.51E-37	postive
HSPA2	AC130371.2	0.547951765	1.46E-43	postive
S100A2	AC130371.2	0.609463958	4.03E-56	postive
VEGFA	AL162586.1	0.500551602	1.60E-35	postive
GMFG	AC147067.1	0.589135248	1.13E-51	postive
FGF9	AL138756.1	0.654334658	3.54E-67	postive
EGF	AL138756.1	0.504048707	4.49E-36	postive
TYMP	U62317.2	0.617767659	4.94E-58	postive
INHBB	LINC00987	0.502059122	9.25E-36	postive
FLT1	LINC00987	0.565251815	7.86E-47	postive
TLR3	AP001372.2	0.515131681	7.30E-38	postive
GMFG	AP004609.3	0.557504909	2.41E-45	postive
S100A2	AP004609.3	0.642429867	4.61E-64	postive
VEGFA	AL139287.1	0.627538371	2.34E-60	postive
VEGFA	C1RL-AS1	0.508859824	7.65E-37	postive
PSMB8	PSMB8-AS1	0.665829856	2.54E-70	postive
IL10RA	PSMB8-AS1	0.558978996	1.27E-45	postive
HLA-DMA	PSMB8-AS1	0.628829735	1.14E-60	postive
CCL5	PSMB8-AS1	0.619541114	1.90E-58	postive
TAP1	PSMB8-AS1	0.652391995	1.17E-66	postive
VEGFA	AL731533.2	0.500228852	1.79E-35	postive
S100A2	RTCA-AS1	0.567152295	3.35E-47	postive
VEGFA	TTC28-AS1	0.629134404	9.59E-61	postive
VEGFA	SNHG20	0.582489888	2.77E-50	postive
FGF9	PLBD1-AS1	0.615219095	1.93E-57	postive
EGF	PLBD1-AS1	0.579345617	1.22E-49	postive
FGF9	AC009779.2	0.564402642	1.15E-46	postive
DEFB1	AC009779.2	0.547182467	2.01E-43	postive
EGF	AC009779.2	0.620704268	1.01E-58	postive
FGF9	AL031123.1	0.611005088	1.80E-56	postive
FGF9	PRDM16-DT	0.67362746	1.56E-72	postive
DEFB1	PRDM16-DT	0.752177396	2.44E-99	postive
EGF	PRDM16-DT	0.62570891	6.47E-60	postive
ESRRG	PRDM16-DT	0.528922153	3.50E-40	postive
ITGB2	AC090559.1	0.629052876	1.00E-60	postive
IL10RA	AC090559.1	0.684320025	1.11E-75	postive
HCK	AC090559.1	0.668688167	4.00E-71	postive

PTPRC	AC090559.1	0.643463516	2.50E-64	postive
TYROBP	USP30-AS1	0.612128416	9.96E-57	postive
ITGB2	USP30-AS1	0.588974548	1.23E-51	postive
PSMB8	USP30-AS1	0.627960273	1.85E-60	postive
IL10RA	USP30-AS1	0.66779314	7.15E-71	postive
HLA-DMA	USP30-AS1	0.681597824	7.21E-75	postive
TYMP	USP30-AS1	0.659499939	1.43E-68	postive
HCK	USP30-AS1	0.514071449	1.09E-37	postive
ISG20	USP30-AS1	0.522992694	3.58E-39	postive
CCL5	USP30-AS1	0.742417985	1.76E-95	postive
TAP1	USP30-AS1	0.712863273	8.60E-85	postive
CXCL9	USP30-AS1	0.596951374	2.41E-53	postive
PTPRC	USP30-AS1	0.548063875	1.39E-43	postive
FCER1G	USP30-AS1	0.629577718	7.48E-61	postive
GMFG	AC012510.1	0.547299417	1.92E-43	postive
S100A2	RPARP-AS1	0.602234846	1.68E-54	postive
VEGFA	AC004148.2	0.534069851	4.47E-41	postive
GMFG	PCAT19	0.619742276	1.70E-58	postive
VEGFA	AC060780.1	0.528200104	4.66E-40	postive
S100A2	AC090425.1	0.545028939	4.97E-43	postive
FGF9	AP000757.1	0.660319161	8.51E-69	postive
DEFB1	AP000757.1	0.712093985	1.56E-84	postive
EGF	AP000757.1	0.586265015	4.55E-51	postive
INHBB	AC007998.3	0.568045914	2.24E-47	postive
S100A2	AC087379.2	0.531569567	1.22E-40	postive
VEGFA	AL354733.3	0.544926467	5.19E-43	postive
FGF9	AL035661.1	0.538351691	7.87E-42	postive
DEFB1	AL035661.1	0.750444701	1.22E-98	postive
EGF	AL035661.1	0.650108979	4.69E-66	postive
VEGFA	AC136475.2	0.548721438	1.05E-43	postive
VEGFA	AC110285.2	0.513294947	1.46E-37	postive
VEGFA	PTOV1-AS2	0.513109752	1.57E-37	postive
VEGFA	AC011472.1	0.59138615	3.78E-52	postive
TYROBP	LINC01094	0.524348495	2.11E-39	postive
ITGB2	LINC01094	0.6225054	3.77E-59	postive
IL10RA	LINC01094	0.502512016	7.85E-36	postive
HCK	LINC01094	0.694963453	5.92E-79	postive
PTPRC	LINC01094	0.609259399	4.49E-56	postive
FCER1G	LINC01094	0.631075013	3.22E-61	postive
ITGB2	AL161785.1	0.522015494	5.24E-39	postive
IL10RA	AL161785.1	0.561708978	3.81E-46	postive
HCK	AL161785.1	0.533248678	6.23E-41	postive
FGF9	MAP4K3-DT	0.551886054	2.73E-44	postive
ITGB2	AC145098.1	0.603356094	9.46E-55	postive

IL10RA	AC145098.1	0.661435515	4.21E-69	postive
HCK	AC145098.1	0.619635202	1.80E-58	postive
PTPRC	AC145098.1	0.508314489	9.36E-37	postive
FCER1G	AC145098.1	0.56411292	1.31E-46	postive
VEGFA	AL022328.2	0.580183023	8.24E-50	postive
FGF9	NNT-AS1	0.539178547	5.61E-42	postive
DEFB1	NNT-AS1	0.512531444	1.95E-37	postive
EGF	NNT-AS1	0.532941028	7.05E-41	postive
ESRRG	NNT-AS1	0.537305935	1.21E-41	postive
VEGFA	ARHGAP27P1-BPTFP1-KPNA2P3	0.509682535	5.64E-37	postive
FGF9	OLMALINC	0.615548876	1.62E-57	postive
DEFB1	OLMALINC	0.595127887	5.97E-53	postive
S100A2	ENTPD3-AS1	0.570689947	6.73E-48	postive
GMFG	LINC01023	0.537117465	1.30E-41	postive
S100A2	LINC01023	0.678368208	6.49E-74	postive
VEGFA	AP000355.1	0.531521775	1.24E-40	postive
FGF9	AL031710.1	0.55633222	4.02E-45	postive
DEFB1	AL031710.1	0.874983578	2.69E-171	postive
EGF	AL031710.1	0.580082056	8.65E-50	postive
FLT1	AC096921.2	0.625505868	7.24E-60	postive
VEGFA	AC005104.1	0.505167753	2.98E-36	postive
C3	LINC01426	0.512584997	1.91E-37	postive
PSMB8	AC242842.1	0.511881996	2.48E-37	postive
VEGFA	RUSC1-AS1	0.519614488	1.32E-38	postive
VEGFA	LENG8-AS1	0.535761725	2.26E-41	postive
IL10RA	AL133371.2	0.530405631	1.94E-40	postive
HCK	AL133371.2	0.503888165	4.76E-36	postive
PTPRC	AL133371.2	0.560859052	5.54E-46	postive
S100A2	AP001505.1	0.52558702	1.30E-39	postive
TLR3	AC098484.1	0.501054353	1.33E-35	postive
VEGFA	AL512791.1	0.507614971	1.21E-36	postive
IL10RA	NCK1-DT	0.540202759	3.68E-42	postive
PTPRC	NCK1-DT	0.574605466	1.11E-48	postive
VEGFA	AC018730.1	0.558381928	1.64E-45	postive
VEGFA	AC097534.2	0.531877489	1.08E-40	postive
FGF9	ZNF710-AS1	0.520398564	9.78E-39	postive
EGF	ZNF710-AS1	0.616498357	9.76E-58	postive
ESRRG	ZNF710-AS1	0.560769058	5.76E-46	postive
VEGFA	AC078864.1	0.519744535	1.26E-38	postive
S100A2	AC244090.1	0.657849056	4.01E-68	postive

Supplementary Table 3

The 29 immune signatures represented by 29 different gene sets.

**aDCs**	CD83, LAMP3, CCL1
**APC co inhibition**	C10orf54, CD274, LGALS9, PDCD1LG2, PVRL3
**APC co stimulation**	CD40, CD58, CD70, ICOSLG, SLAMF1, TNFSF14, TNFSF15, TNFSF18, TNFSF4, TNFSF8, TNFSF9
**B cells**	BACH2, BANK1, BLK, BTLA, CD79A, CD79B, FCRL1, FCRL3, HVCN1, RALGPS2
**CCR**	CCL16, TPO, TGFBR2, CXCL2, CCL14, TGFBR3, IL11RA, CCL11, IL4I1, IL33, CXCL12, CXCL10, BMPER, BMP8A, CXCL11, IL21R, IL17B, TNFRSF9, ILF2, CX3CR1, CCR8, TNFSF12, CSF3, TNFSF4, BMP3, CX3CL1, BMP5, CXCR2, TNFRSF10D, BMP2, CXCL14, CCL28, CXCL3, BMP6, CCL21, CXCL9, CCL23, IL6, TNFRSF18, IL17RD, IL17D, IL27, CCL7, IL1R1, CXCR4, CXCR2P1, TGFB1I1, IFNGR1, IL9R, IL1RAPL1, IL11, CSF1, IL20RA, IL25, TNFRSF4, IL18, ILF3, CCL20, TNFRSF12A, IL6ST, CXCL13, IL12B, TNFRSF8, IL6R, BMPR2, IFNE, IL1RAPL2, IL3RA, BMP4, CCL24, TNFSF13B, CCR4, IL2RA, IL32, TNFRSF10C, IL22RA1, BMPR1A, CXCR5, CXCR3, IFNA8, IL17REL, IFNB1, IFNAR1, TNFRSF1B, CCL17, IFNL1, IL16, IL1RL1, ILK, CCL25, ILDR2, CXCR1, IL36RN, IL34, TGFB1, IFNG, IL19, ILKAP, BMP2K, CCR10, ILDR1, EPO, CCR7, IL17C, IL23A, CCR5, IL7, EPOR, CCL13, IL2RG, IL31RA, TNFAIP6, IFNL2, BMP1, IL12RB1, TNFAIP8, IL4R, TNFRSF6B, TNFAIP8L1, TNFRSF10B
**CD8+ T cells**	CD8A
**Check-point**	*IDO1, LAG3, CTLA4, TNFRSF9, ICOS, CD80, PDCD1LG2, TIGIT, CD70, TNFSF9, ICOSLG, KIR3DL1, CD86, PDCD1, LAIR1, TNFRSF8, TNFSF15, TNFRSF14, IDO2, CD276, CD40, TNFRSF4, TNFSF14, HHLA2, CD244, CD274, HAVCR2, CD27, BTLA, LGALS9, TMIGD2, CD28, CD48, TNFRSF25, CD40LG, ADORA2A, VTCN1, CD160, CD44, TNFSF18, TNFRSF18, BTNL2, C10orf54, CD200R1, TNFSF4, CD200, NRP1*
**Cytolytic activity**	*PRF1, GZMA*
**DCs**	*CCL17, CCL22, CD209, CCL13*
**HLA**	*HLA-E, HLA-DPB2, HLA-C, HLA-J, HLA-DQB1, HLA-DQB2, HLA-DQA2, HLA-DQA1, HLA-A, HLA-DMA, HLA-DOB, HLA-DRB1, HLA-H, HLA-B, HLA-DRB5, HLA-DOA, HLA-DPB1, HLA-DRA, HLA-DRB6, HLA-L, HLA-F, HLA-G, HLA-DMB, HLA-DPA1*
**Inflammation-promoting**	*CCL5, CD19, CD8B, CXCL10, CXCL13, CXCL9, GNLY, GZMB, IFNG, IL12A, IL12B, IRF1, PRF1, STAT1, TBX21*
**Macrophages**	*C11orf45, CD68, CLEC5A, CYBB, FUCA1, GPNMB, HS3ST2, LGMN, MMP9, TM4SF19*
**MHC class I**	*B2M, HLA-A, TAP1*
**Neutrophils**	*EVI2B, HSD17B11, KDM6B, MEGF9, MNDA, NLRP12, PADI4, SELL, TRANK1, VNN3*
**NK cells**	*KLRC1, KLRF1*
**Parainflammation**	*CXCL10, PLAT, CCND1, LGMN, PLAUR, AIM2, MMP7, ICAM1, MX2, CXCL9, ANXA1, TLR2, PLA2G2D, ITGA2, MX1, HMOX1, CD276, TIRAP, IL33, PTGES, TNFRSF12A, SCARB1, CD14, BLNK, IFIT3, RETNLB, IFIT2, ISG15, OAS2, REL, OAS3, CD44, PPARG, BST2, OAS1, NOX1, PLA2G2A, IFIT1, IFITM3, IL1RN*
**pDCs**	*CLEC4C, CXCR3, GZMB, IL3RA, IRF7, IRF8, LILRA4, PHEX, PLD4, PTCRA*

**T cell co-inhibition**	*BTLA, C10orf54, CD160, CD244, CD274, CTLA4, HAVCR2, LAG3, LAIR1, TIGIT*
**T cell co-stimulation**	CD2, CD226, CD27, CD28, CD40LG, ICOS, SLAMF1, TNFRSF18, TNFRSF25, TNFRSF4, TNFRSF8, TNFRSF9, TNFSF14
**T helper cells**	CD4
**Tfh**	PDCD1
**Th1 cells**	IFNG, TBX21, CTLA4, STAT4, CD38, IL12RB2, LTA, CSF2
**Th2 cells**	PMCH, LAIR2, SMAD2, CXCR6, GATA3, IL26
**TIL**	ITM2C, CD38, THEMIS2, GLYR1, ICOS, F5, TIGIT, KLRD1, IRF4, PRKCQ, FCRL5, SIRPG, LPXN, IL2RG, CCL5, LCK, TRAF3IP3, CD86, MAL, LILRB1, DOK2, CD6, PAG1, LAX1, PLEK, PIK3CD, SLAMF1, XCL1, GPR171, XCL2, TBX21, CD2, CD53, KLHL6, SLAMF6, CD40, SIT1, TNFRSF4, CD79A, CD247, LCP2, CD3D, CD27, SH2D1A, FYB, ARHGAP30, ACAP1, CST7, CD3G, IL2RB, CD3E, FCRL3, CORO1A, ITK, TCL1A, CYBB, CSF2RB, IKZF1, NCF4, DOCK2, CCR2, PTPRC, PLAC8, NCKAP1L, IL7R, 6-Sep, CD28, STAT4, CD8A, LY9, CD48, HCST, PTPRCAP, SASH3, ARHGAP25, LAT, TRAT1, IL10RA, PAX5, CCR7, DOCK11, PARVG, SPNS1, CD52, HCLS1, ARHGAP9, GIMAP6, PRKCB, MS4A1, GPR18, TBC1D10C, GVINP1, P2RY8, EVI2B, VAMP5, KLRK1, SELL, MPEG1, MS4A6A, ARHGAP15, MFNG, GZMK, SELPLG, TARP, GIMAP7, FAM65B, INPP5D, ITGA4, MZB1, GPSM3, STK10, CLEC2D, IL16, NLRC3, GIMAP5, GIMAP4, IFFO1, CFH, PVRIG, CFHR1
**Treg**	IL12RB2, TMPRSS6, CTSC, LAPTM4B, TFRC, RNF145, NETO2, ADAT2, CHST2, CTLA4, NFE2L3, LIMA1, IL1R2, ICOS, HSDL2, HTATIP2, FKBP1A, TIGIT, CCR8, LTA, SLC35F2, IL21R, AHCYL1, SOCS2, ETV7, BCL2L1, RRAGB, ACSL4, CHRNA6, BATF, LAX1, ADPRH, TNFRSF4, ANKRD10, CD274, CASP1, LY75, NPTN, SSTR3, GRSF1, CSF2RB, TMEM184C, NDFIP2, ZBTB38, ERI1, TRAF3, NAB1, HS3ST3B1, LAYN, JAK1, VDR, LEPROT, GCNT1, PTPRJ, IKZF2, CSF1, ENTPD1, TNFRSF18, METTL7A, KSR1, SSH1, CADM1, IL1R1, ACP5, CHST7, THADA, CD177, NFAT5, ZNF282, MAGEH1
**Type I IFN Reponse**	DDX4, IFIT1, IFIT2, IFIT3, IRF7, ISG20, MX1, MX2, RSAD2, TNFSF10
**Type II IFN Reponse**	GPR146, SELP, AHR
**iDCs**	CD1A, CD1E
**Mast cells**	CMA1, MS4A2, TPSAB1
